# Role of backbone strain in de novo design of complex α/β protein structures

**DOI:** 10.1038/s41467-021-24050-7

**Published:** 2021-06-24

**Authors:** Nobuyasu Koga, Rie Koga, Gaohua Liu, Javier Castellanos, Gaetano T. Montelione, David Baker

**Affiliations:** 1 University of Washington, Department of Biochemistry and Howard Hughes Medical Institute, Seattle, Washington, WA USA; 2 Research Center of Integrative Molecular Systems, Institute for Molecular Science, National Institutes of Natural Sciences, Okazaki, Aichi Japan; 3 Protein Design Group, Exploratory Research Center on Life and Living Systems (ExCELLS), National Institutes of Natural Sciences, Okazaki, Aichi Japan; 4 SOKENDAI, The Graduate University for Advanced Studies, Hayama, Kanagawa Japan; 5 Nexomics Biosciences, Rocky Hill, NJ USA; 6 Department of Chemistry and Chemical Biology, and Center for Biotechnology and Interdisciplinary Sciences, Rensselaer Polytechnic Institute, Troy, New York, NY USA

**Keywords:** Structural biology, Solution-state NMR, Computational biophysics, Protein design

## Abstract

We previously elucidated principles for designing ideal proteins with completely consistent local and non-local interactions which have enabled the design of a wide range of new αβ-proteins with four or fewer β-strands. The principles relate local backbone structures to supersecondary-structure packing arrangements of α-helices and β-strands. Here, we test the generality of the principles by employing them to design larger proteins with five- and six- stranded β-sheets flanked by α-helices. The initial designs were monomeric in solution with high thermal stability, and the nuclear magnetic resonance (NMR) structure of one was close to the design model, but for two others the order of strands in the β-sheet was swapped. Investigation into the origins of this strand swapping suggested that the global structures of the design models were more strained than the NMR structures. We incorporated explicit consideration of global backbone strain into the design methodology, and succeeded in designing proteins with the intended unswapped strand arrangements. These results illustrate the value of experimental structure determination in guiding improvement of de novo design, and the importance of consistency between local, supersecondary, and global tertiary interactions in determining protein topology. The augmented set of principles should inform the design of larger functional proteins.

## Abstract (web summary)

The authors show that consideration of global backbone strain enables successful de novo design of larger αβ-proteins with five- and six- stranded β-sheets flanked by α-helices.

## Introduction

Protein design provides an opportunity to test our understanding of protein folding and investigate how amino acid sequences determine unique folded structures^1–17^. There has been considerable progress in de novo protein design, stemming in part from the elucidation of principles^6,18^ for designing ideal protein structures^19^ stabilized by consistent local and nonlocal interactions. These principles are embodied in a set of design rules relating local backbone structures to supersecondary structure packing of α-helices on paired β-strands, which generate funnel-shaped energy landscapes by disfavoring non-native states^6,9^. The principles have made possible the de novo design of a range of ideal protein structures, including four-stranded αβ-proteins with different topologies^6^, sizes and shapes^9^, and larger TIM-barrels^12^.

Most functional sites in proteins are composed of multiple structural elements distant along the linear sequence. For example, enzymes often have active sites containing catalytic residues with adjacent substrate binding pockets formed by different parts of the structure. This coming together in three dimensions of parts of the protein distant along the sequence has the advantage of allowing a much broader range of geometries than possible in a local chain segment, and the enclosing of binding sites on nearly all sides. The core of many enzymes is composed of a central β-sheet with five or more strands surrounded on both sides by α-helices; the ideal αβ-proteins we designed previously, with the exception of the TIM barrel, are too small to harbor active sites. To access more of protein functional space, and to stringently test our understanding of the sequence dependence of protein folding, we sought to design larger αβ-proteins consisting of five or six β-strands flanked on both sides by α-helices.

In this work, we test the generality of our design principles by applying them to the de novo design of larger αβ-proteins. One class of these designs folds into topologies different from the computational models, with the order of strands in the β-sheet swapped. Investigation into the origins of this strand swapping revealed that the design principles must be extended to incorporate explicit consideration of global backbone strain to provide control over folded topologies for larger αβ-proteins.

## Results

### Design of five- and six- strand αβ-proteins

**Fig. 1:**
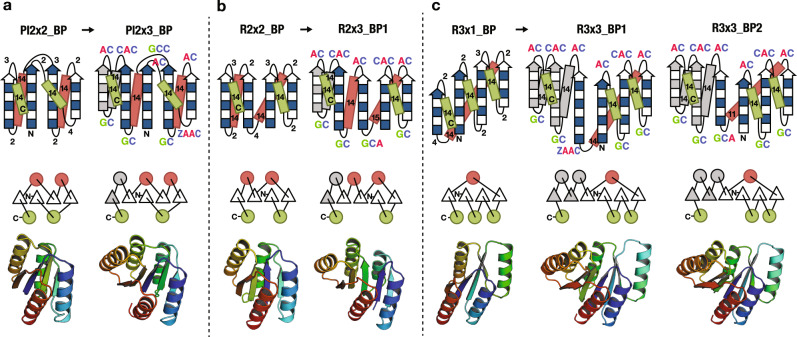
Backbone blueprints and design models for target folds. (top) Backbone blueprints for **a** P-loop2×3-fold: Pl2×3_BP, **b** Rossmann2×3-fold: R2x3_BP1, **c** Rossmann3×3-fold: R3×3_BP1 and R3x3_BP2. Helix lengths are represented by numbers within green and red rectangles, strand residues indicated by filled and open boxes: filled boxes represent pleats (the vectors from Cα atom to Cβ atom) coming out of the page and the open boxes represent pleats going into the page. Loops are labeled by the extended ABEGO torsion patterns (see Methods and Supplementary Fig. 1). (middle) Design topologies are illustrated with circles (helices) and triangles (strands) connected by solid lines (loops). (bottom) Design models created from the blueprints (top). The blueprints were created by inserting an αβ or αβ-αβ motif (gray color) into the position immediately before the C-terminal helix in the blueprints we used previously to design four-stranded β-sheet proteins^6^. The secondary structure lengths and the loop ABEGO patterns are based on the design rules for βαβ-motif described in Fig. S5 and S6 of ref. ^9^, with the extension of the ABEGO bins. For Rossmann3×3-fold, we experimented with two blueprints, changing the register shift and the length of the fourth strand.

We selected as design targets two topologies which are widespread in enzymes in nature: the P-loop fold and the Rossmann fold. The two are similar, but have permuted orders of the β-strands in the central β-sheet (Fig. 1). We built structure blueprints for the P-loop and Rossmann folds with five-stranded β-sheets flanked by five helices, and for a six-stranded Rossmann fold flanked by six helices (three on each side) by extending those for the previously designed four-stranded proteins, the P-loop2×2- and Rossmann2×2- folds^6^ (Fig. 1; for the six-stranded Rossmann design, we experimented with two blueprints). For each blueprint, we carried out Rosetta sequence-independent folding simulations^6^ to generate backbone structures (see Backbone building in Methods and Supplementary Figs. 1, 2) and subsequently Rosetta full-atom sequence design calculations^5^ to build side chains on each of the generated backbone structures (see Methods). Designs with low energy^20^, tight side chain packing^21^, and high compatibility between local sequence and structure^6^ were selected, and their energy landscapes were mapped using Rosetta de novo folding simulations^20^. Designs with sequences having funneled energy landscapes leading into the designed structure were selected for experimental characterization.

**Fig. 2:**
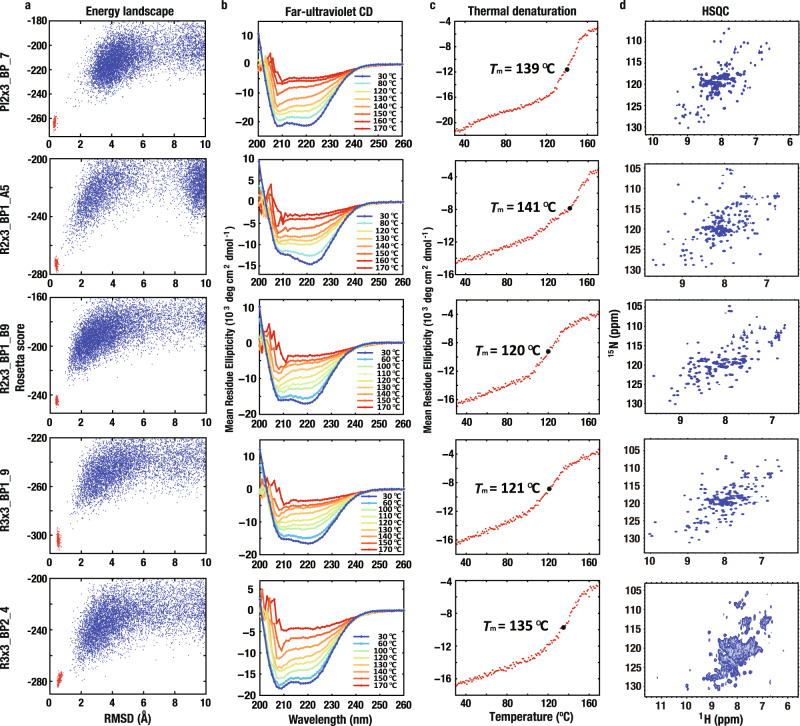
Design characterization. **a** Energy landscapes from Rosetta ab initio structure prediction simulations. Blue points represent the lowest energy structures obtained in independent Monte Carlo structure prediction trajectories starting from an extended chain for each sequence; the y-axis is the Rosetta all atom energy, the x-axis, the root mean square deviation (RMSD) to the design model. Red points represent the lowest energy structures obtained in trajectories starting from the design model. **b** Far-ultraviolet circular dichroism (CD) spectra at temperatures up to 170 °C and **c** Thermal denaturation curves at 222 nm with the transition midpoint temperature, ${T}_{m}$. **d** Two-dimensional ^1^H-^15^N HSQC spectra at 25 °C and 600 MHz.

We obtained synthetic genes encoding 12 designs for Pl2×3_BP, 31 for R2×3_BP1, 12 for R3×3_BP1, and 16 for R3×3_BP2 (Supplementary Table 11, R2×3_BP1_A and R2×3_BP1_B designs were made with slightly different computational protocols; see Methods). Some designs (Pl2×3_BP: 1, R2×3_BP1_A: 1, R2×3_BP1_B: 3, R3×3_BP1: 6, and R3×3_BP2: 4) have weak sequence similarity to proteins of unknown structure in the nr database (Blast E value <0.005), but the remainder do not have detectable similarity to naturally occurring proteins. The designed proteins were expressed, purified, and characterized by circular dichroism (CD) spectroscopy, size-exclusion chromatography combined with multi-angle light scattering (SEC-MALS), and ^1^H-^15^N heteronuclear single quantum coherence (HSQC) NMR spectroscopy. For all target folds, 56 of 71 designed proteins were found to be expressed and highly soluble, and have CD spectra typical of αβ-proteins from room temperature to ~100 °C; more than half of those were found to be monomeric by SEC-MALS (Supplementary Tables 2–4, 6, and 7). However, only a minority of the designs had well-dispersed sharp NMR peaks; for R3x3_BP2 designs, none did (Fig. 2, and Supplementary Tables 2–4, 6, and 7). The experimental results for all designs for all target folds are summarized in Supplementary Table 1.

**Fig. 3:**
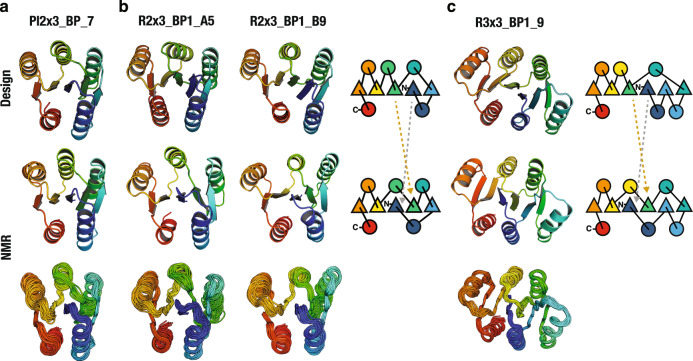
NMR structure determination reveals strand swapping. Design models (upper row), together with a representative conformer (middle row) and the ensemble of conformers (lower row) determined by NMR, for **a** P-loop fold: Pl2×3_BP_7 (PDB: 5GAJ), **b** Rossmann2×3 fold: R2×3_BP1_A5 (2L69) and R2×3_BP1_B9 (2LCI), and **c** Rossmann3×3 fold: R3×3_BP1_9 (2L82); topology diagrams are on the right. As is clear from the topology diagrams, in the NMR structures for the Rossmann2×3- and 3×3- folds, the positions of the green and blue β-strands are swapped compared to the order in the design models.

One monomeric design with well-dispersed sharp NMR peaks for each of Pl2×3_BP and R3×3_BP1: Pl2×3_BP_7 and R3×3_BP1_9, and one for each of the two design cycles for R2×3_BP1: R2×3_BP1_A5 and R2×3_BP1_B9 (the sequence identity between the two is 28%) were selected for NMR structure determination (Fig. 2 and Supplementary Fig. 17). The NMR structure of the P-loop fold (Pl2×3_BP_7) was close to the computational design model with average root mean square deviation (RMSD) of Cα atoms of 1.1 Å (Fig. 3, Supplementary Fig. 4, and Supplementary Tables 9, 10). Surprisingly, however, while the NMR structures of the Rossmann folds (R2×3_BP1_A5, R2×3_BP1_B9, and R3×3_BP1_9) had the designed three-layer αβα-sandwich architecture, the order of the β-strands was swapped, resulting in the P-loop topology (Fig. 3, Supplementary Fig. 5, and Supplementary Table 10). The Rosetta energies for the computational models are lower than those for the relaxed NMR structures for all three strand-swapped designs (Supplementary Fig. 6). The strand-swapping was observed for each of the three Rossmann fold design attempts, implying that it was not an aberration, but reflected some fundamental shortcomings in the energy function and/or our design concepts.

### Negative design does not solve strand swapping

**Fig. 4:**
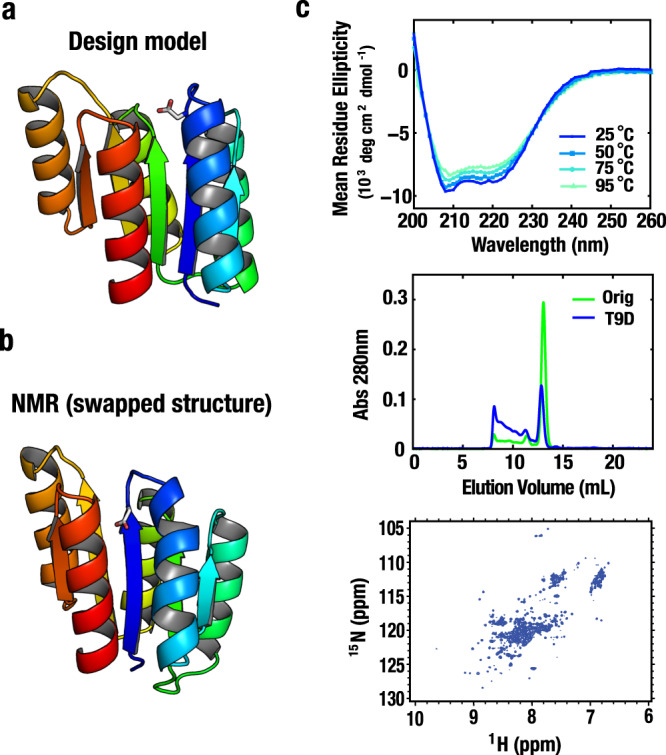
Strand-swapped structure is robust to negative design. The mutation T9D in **a** the design model and **b** the NMR structure of R2×3_BP1_B9. Both mutated structures were generated by using Foldit^53^ followed by Rosetta minimization^25^. **c** (top) Temperature dependence of the CD spectra of the T9D mutant. (middle) Size exclusion chromatograms at 280 nm of the original R2×3_BP1_B9 (Orig) and the T9D mutant. (bottom) Two-dimensional ^1^H-^15^N HSQC spectrum of the T9D mutant at 25 °C and 600 MHz.

The Rossmann fold design model conformations and the observed strand swapped P-loop conformations could have roughly similar free energies, with the latter favored due to kinetic accessibility or a small free energy advantage. Alternatively, the observed P-loop conformation could be substantially lower in free energy despite the predictions of the Rosetta energy model. To distinguish between these possibilities, we introduced a negative design element that strongly disfavors the swapped P-loop strand ordering. Following visual inspection, residue Thr9 in R2x3_BP1_B9 was mutated to Asp, which is expected to disfavor the strand-swapped state as the charged residue would become buried (Fig. 4a, b). The Thr9Asp protein was found to be in a molten globule state^22^ (Fig. 4c), suggesting the possibility that the Rossmann designs have a single free energy minimum only at the strand-swapped state.

### Compatibility of blueprint with global tertiary structure

**Fig. 5:**
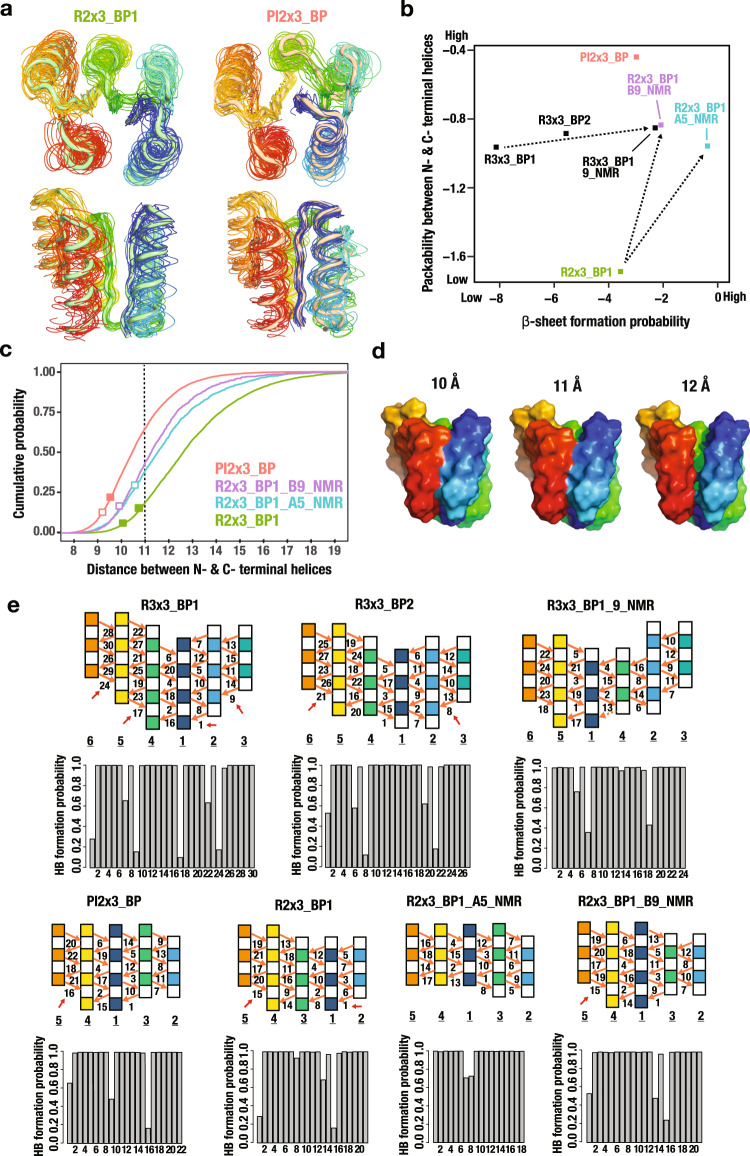
Increased structural frustration in backbone ensembles generated from original design blueprints. **a** Backbone structure ensembles generated from blueprints for R2×3_BP1 and Pl2×3_BP. Individual members of the ensembles are represented by wireframe and averages over the thousands of backbones generated for each blueprint, in tubes. **b** The β-sheet formation probability and packability between the N- and C- terminal helices for the blueprints used in the designs and the strand-swapped NMR-structure-based blueprints. **c** Cumulative probability distributions of the distance between the N- and C- terminal helices for Pl2×3_BP, R2×3_BP1_A5_NMR, R2×3_BP1_B9_NMR, and R2×3_BP1. Filled boxes represent the distances of design models and open boxes, those of NMR structures. **d** Structures generated from R2×3_BP1 with the distance between the N- and C- terminal helices 10, 11, and 12 Å demonstrate that close helix packing is achieved at less than ~11 Å (for visualization, valine side chains are placed on the backbones at all residue positions). **e** (top) Schematic diagrams of hydrogen bonds in the parallel β-sheets of the designs and NMR structures. (bottom) Hydrogen-bond (HB) formation probability for each hydrogen bond (The numbering in the bar graph corresponds to that shown in the β-sheet schematics). Red arrows show the incomplete hydrogen bonds, in which donor is provided by the first residue of the strands. The dotted hydrogen bond in R3×3_BP1_9_NMR was not included because this bond is broken both in the NMR structures and the simulation ensemble.

The swapped P-loop fold observed in the NMR structure could be lower in free energy either because of specific sidechain–sidechain interactions around the swapped strands being suboptimal in the design model, or because of more global energetic strain in the backbone in the original design configuration. We considered the second possibility more likely because the two strands which swap are internal to the β-sheet, and hence have very similar patterns of hydrophobic residues; the sidechain–sidechain interactions in the design model and the NMR structure are thus similar. To investigate possible backbone strain in the design model topology, we carried out Rosetta sequence-independent folding simulations^6^ to generate backbone structure ensembles (Fig. 5a, see Backbone building in Methods) for the design model blueprints (Supplementary Fig. 2) and blueprints corresponding to the NMR structure (Supplementary Fig. 7), which are different in strand lengths as well as strand order: the strand lengths of the NMR structures are generally shorter than those of the design models (Fig. 5e). We then analyzed the extent of hydrogen bonding between β-strands in the β-sheet (β-sheet formability), and the packing between the N- and C- terminal helices that zip up the folded structures. The β-sheet formation probability was evaluated as the sum of the log of the probability in the ensemble of each β-sheet hydrogen-bond, and the packability of the terminal helices, as the log of the probability in the ensemble of the two helices close enough for the side chains to pack (see Methods and Supplementary Fig. 8). We interpret the extent of formation of the β-sheet and packing of the helices as a measure of the overall strain associated with the backbone topology: in more strained arrangements there is more likely to be more frustration in achieving these properties than in less strained arrangements.

There was a clear difference in both measures between the original design blueprint and the experimentally observed blueprint—the former gives rise to poorer β-strand hydrogen bonding and packing between the terminal helices (Fig. 5b; the arrows connect the values for the original design models with those of the NMR structures). For the Rossmann2×3-fold, in the backbone ensemble generated from the R2×3_BP1 blueprint used in the design, the median distance between the terminal helices was 12.6 Å, whereas in the ensembles generated from the blueprints observed in the NMR structures (R2×3_BP1_A5_NMR (2l69) and R2×3_BP1_B9_NMR (2lci)), the terminal helices are on average 11.4 Å apart (Fig. 5c, d and Supplementary Fig. 9). The β-sheet hydrogen bonding was also more disrupted in the ensembles for the original design model blueprints (Fig. 5e; shorter vertical black bars indicate incomplete hydrogen bond formation), particularly the hydrogen bonds made by the first residues in the β-strands (red arrows in Fig. 5e). This is likely due to the inherent twisting of parallel β-strands, which when the strands are longer leads to separations at their ends that are beyond hydrogen bonding distance (Note that the hydrogen bonds made by the last strand residues are frequently broken). For the Rossmann3×3-fold, β-sheet hydrogen bond frustration in the structure ensembles generated from the R3×3_BP1 and R3×3_BP2 design blueprints is relieved in the ensemble generated from the R3×3_BP1_9_NMR (2l82) based blueprint in part because the swapped strands become shorter (Fig. 5e).

Comparison of the R2×3_BP1 computational design models to the backbone ensemble generated from the design blueprint revealed that the closer packing of the terminal helices in the former arose from strong bending of the β-sheet and/or local backbone distortion of the first helix against the β-sheet (Supplementary Figs. 10, 11). These backbone distortions likely result from iteration between sequence design and energy minimization of the entire structure, perhaps to bring the terminal helices closer together. In contrast, the β-sheet geometry in the NMR structure was close to that observed in the corresponding backbone ensemble (Supplementary Fig. 12). We speculated that the strand swapping observed in the NMR structures arose because it allows the terminal helices to come close for good sidechain–sidechain packing without requiring energetically unfavorable β-sheet bending or local backbone distortion. The β-strands to which the N- and C- terminal helices are attached, the first and last β-strands, are closer together in the NMR (P-loop) blueprints than in the design model (Rossmann) blueprint as they are separated by one intervening β-strand rather than two (see Fig. 3b). The incomplete β-sheet hydrogen bond formation in the ensembles generated from the design model blueprints (Fig. 5e) suggests that even with the helices further apart, there is strain in the design model β-sheets that is released upon strand swapping.

These results highlight a blind spot in our original design strategy^6,9^. The rules we developed, which relate local backbone structures to supersecondary structure motifs involving two or three secondary structure elements, reduce local backbone strain, but do not address overall backbone strain, which emerges only at the level of the entire tertiary structure. For success in controlling protein structure in de novo design, overall global backbone strain must be considered. Guided by these observations, we next explored the design of blueprints capable of generating the Rossmann fold without strand swapping, by achieving consistency between local, supersecondary structure, and global tertiary interactions.

### Explore frustration-free blueprints

**Fig. 6:**
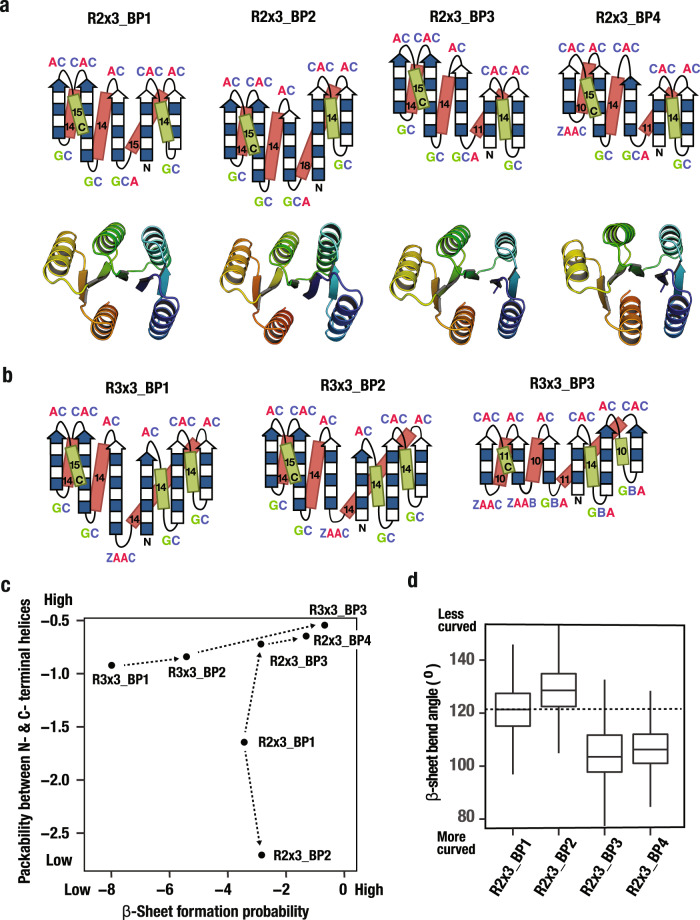
Exploration of frustration-free blueprints. **a** Alternative backbone blueprints for the Rossmann2×3-fold, together with the averaged structures over the backbone ensembles generated from the blueprints. **b** Alternative backbone blueprints for the Rossmann3×3-fold. **c** β-sheet formation probability and packability between the N- and C- terminal helices for each blueprint. **d** β-sheet bend angle for each Rossmann2×3-fold blueprint (box plots: horizontal lines indicate median and upper and lower quartiles (Q_3/4_ and Q_1/4_), respectively; vertical lines extend to Q_3/4_ + 1.5 x (Q_3/4_ − Q_1/4_) and Q_1/4_ − 1.5 x (Q_3/4_ − Q_1/4_). *n* = 6174, 6136, 6768, and 8056 independently generated backbone structures for R2×3_BP1, R2×3_BP2, R2×3_BP3, and R2×3_BP4.

To obtain frustration-free blueprints for the Rossmann2×3-fold, improvement of the packability of the terminal helices is required as is obvious from Fig. 5a–d. For this purpose, we attempted to build a more curved β-sheet by introducing strand register shifts. To investigate the relation of strand register shift to the curvature of the β-sheet, backbone ensembles were generated for blueprints that have negative (R2×3_BP2) and positive (R2×3_BP3) register shifts between the first and the third strands compared with R2×3_BP1 (Fig. 6a). The backbone ensembles have more curved β-sheets for R2×3_BP3 and less curved for R2×3_BP2 compared with R2×3_BP1 (Fig. 6d). The registry dependent rigid-body packing orientation of the C-terminal half of the designs (β_3_α_3_β_4_α_4_β_5_α_5_) relative to the N-terminal half (β_1_α_1_β_2_) generates different β-sheet curvatures (Supplementary Fig. 13). The internal twisting of each strand is independent of this registry, and hence does not contribute to curvature. As expected, the helix packability for R2×3_BP3 is improved, and that of R2×3_BP2 is worse (Fig. 6c and Supplementary Fig. 14). To increase β-strand hydrogen bonding and reduce β-sheet frustration, the strand lengths in R2×3_BP3 were shortened to generate R2×3_BP4 (Fig. 6a and Supplementary Fig. 15). The resulting backbone ensembles showed significantly reduced frustration both in β-sheet formation and helix packability compared with R2×3_BP1 (Fig. 6c). To obtain frustration-free blueprints for the Rossmann3×3-fold, alleviation of the β-sheet frustration is even more important (Fig. 5b): β-sheet formation is frustrated in R3×3_BP1 and only slightly relieved for R3×3_BP2 by shortening the fourth strand length (Fig. 6b). To further reduce this frustration, the lengths of the β-strands were shortened to generate R3×3_BP3 (Fig. 6b and Supplementary Fig. 15). The resulting ensemble was almost frustration-free (Fig. 6c).

### Experimental characterization of Rossmann2×3- and 3×3- designs created from frustration-free blueprints

**Fig. 7:**
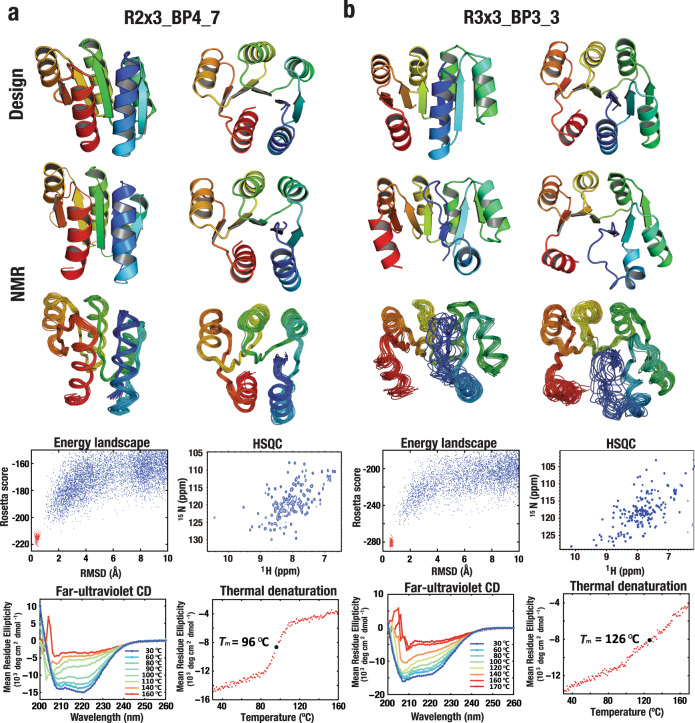
Success in Rossmann-fold design using frustration-free blueprints. NMR structures and design models are represented as in Fig. 3, and biophysical characterization and panel descriptions are as in Fig. 2. **a** R2×3_BP4_7 (PDB: 6XEH) and **b** R3×3_BP3_3 (7KBQ).

We used these frustration-resolving R2×3_BP4 and R3×3_BP3 blueprints to guide a second round of full side chain design and experimental characterization. We obtained synthetic genes encoding eight designs for R2×3_BP4 and ten for R3×3_BP3 (Supplementary Table 11, the sequences of the R2×3_BP4 and one of the R3×3_BP3 have weak (Blast E value <0.005) sequence similarity to natural proteins of unknown structure). The proteins were expressed, purified, and characterized by CD spectroscopy, SEC-MALS, and ^1^H-^15^N HSQC NMR spectroscopy. For the Rossmann2×3-fold, all designs are well expressed and highly soluble, and all but one design show CD spectra characteristic of αβ-proteins (Supplementary Table 5). Six out of the eight designs were found to be monomeric by SEC-MALS and five show well-dispersed sharp NMR peaks (Supplementary Table 5; the summary is shown in Supplementary Table 1). For one design that was monomeric and had the αβ-protein CD spectrum and the expected number of well-dispersed sharp NMR peaks (R2×3_BP4_7), the solution NMR structure was determined (Fig. 7a and Supplementary Fig. 17; for Rosetta energy comparison between the design and NMR models, Supplementary Fig. 16). The resulting solution NMR structure has a correct strand-order and agrees closely with the computational design model (Fig. 7a and Supplementary Tables 9, 10).

For the Rossmann3×3-fold, all the designs were expressed, and half (five designs) were soluble and had the expected αβ-protein CD spectra (Supplementary Table 8). Four were monomeric and two had well-dispersed and sharp NMR peaks (Supplementary Table 8; the summary is shown in Supplementary Table 1). For one design that had sharper NMR peaks (R3x3_BP3_3), we determined the solution NMR structure (Fig. 7b and Supplementary Figs. 16, 17). The NMR structure has the designed strand-order of the Rossmann3×3-fold, but the conformation of the N-terminal helix is not well-defined (Fig. 7b and Supplementary Tables 9, 10), likely due to conformational dynamics. The NMR spectra also show some residues with multiple resonances, due to slow exchange between multiple conformations in dynamic equilibrium. While the revisions to the design blueprint succeeded in achieving the target Rossmann3×3-fold, control over the dynamic structural details of a complex structure of this size (126 residues, six strands and six helices with, in contrast the TIM barrel, little internal symmetry) remains challenging.

## Discussion

Our previously described protein design principles were based on consideration of the backbone strain that arises when secondary structural elements are assembled into supersecondary structure motifs with helices packed on paired β-strands using loop connections of different lengths. As shown here, these principles, while sufficient for accurately designing folds with four and fewer β-strands, failed to accurately define strand order in more complex structures with five- and six- stranded β-sheets. The strand-swapping observed in the first three Rossman-fold design attempts suggested something was missing in our energy function and/or design concepts; the puzzle was further highlighted by the failure of negative design efforts disfavoring the strand-swapped state to restore the designed strand-order, suggesting the designs have a single free energy minimum at the swapped state. We considered a number of possible explanations for the strand swapping, and carrying out experiments to investigate these, and through iteration between computational design and experiment established that long-range backbone strain likely accounts for the favoring of the swapped state, as described in the following paragraph. This resolution of the original strand swapping puzzle highlights the critical contribution of experimental structure determination to iterative improvement of computational protein design methodology.

Our results suggest that control over strand order requires consideration not only of local backbone strain associated with supersecondary structure formation, but also backbone strain arising from incompatibility between a global tertiary structure and the constituent supersecondary structure elements. The differences in β-sheet hydrogen bond formation probability, and in packing between the N- and C- terminal helices, in backbone ensembles generated using blueprints for the design models and the observed NMR structures suggest lower backbone strain in the experimentally observed strand arrangement than the original designed one. Modulating the design blueprints to relieve frustration in β-sheet formation by shortening strand lengths, and to increase helix packability by making the β-sheet curve through strand register shifting, resulted in designs that fold into the original target Rossmann topologies. Our results suggest that the global strain associated with a given blueprint-topology combination must be taken into account to accurately determine the topologies of designed proteins. In this work, the low strain backbone blueprints were identified by trial-and-error exploration (Fig. 6), moving forward it should be possible to automate this search.

The failure of the Rosetta energy calculations to identify the strand swapped P-loop topology as the lowest free energy state for the original designs may be due to both energetic and entropic factors. First, the energy function may inaccurately capture the cost of bending the β-sheet to bring the N- and C- terminal strands together in the original design models; indeed, the design models have lower Rosetta energy than the observed NMR structures. The exact defects in the energy function are hard to identify because of compensation as the errors may be spread throughout the structure (the sheet can either stay flat and the helices pack less well, or bend to allow helices to pack, or everything in between—each gives rise to a different energy signature). Second, the strain could result in a decrease in configurational entropy in the original Rossmann topology—the ensemble of accessible low energy structures may be larger for the swapped strand arrangement because there are many more ways for the terminal helices to come together for close side chain packing without distorting the β-sheet (as noted before, the strands to which they are attached are closer in the β-sheet after the swap). This is supported by our backbone ensemble calculations, which reveal a much smaller population of energetically frustrated conformations for the strand swapped conformation (Supplementary Fig. 9). Our backbone ensembles in a sense provide a simple readily computable proxy for configurational entropy, which is notoriously difficult to compute for large proteins.

Our results suggest that incorporation of locally nonideal features to relieve strain at the global level can be necessary even with some cost of local frustration (structural suboptimality). Naturally occurring proteins likely relieve backbone strain by shortening strand lengths and making curved sheets with strand register shifts, but also incorporate longer loops and other nonideal features which not only play roles in function but also help release structural frustration in β-sheet formation and helix packing. Indeed, in design of curved β-sheets without frustration, incorporation of deviations from ideality such as β-bulges and glycine in the β-strands (glycine kinks) has been found to be important for releasing strain^15^. Our results suggest that consideration of overall backbone strain should likewise allow the de novo design of larger αβ-proteins with more complex functional sites.

## Methods

### Backbone building

(Step 1) The backbone structures for each blueprint were built part by part (Supplementary Fig. 2) by carrying out the Rosetta sequence-independent folding simulations using a coarse-grained model, in which each residue is represented by main chain atoms (N, NH, Cα, C, and CO) and a side chain pseudo atom^6,9^. The goal of this step is to build “rough” backbone structures with a target topology. Therefore, the secondary structures specified in the blueprint are not required to be completely formed. In the simulations, the backbone structures were built from the N-terminal part, and then the rest of parts were built on top of the built structure after confirming that this contains no helix kink and forms the β-sheet in which each strand consists of at least one residue and all strands have the register designated by the blueprint (secondary structures were defined by DSSP^23^); this loose β-sheet formation criterion was used for preventing parts from being locally optimized. The Rosetta potential function used in the simulations includes steric repulsion (vdw = 1.0), overall compaction (rg = 1.0), secondary structure pairings (ss_pair = 1.0, rsigma = 1.0, and hs_pair = 1.0), and main-chain hydrogen bonds (hbond_sr_bb = 1.0 and hbond_lr_bb = 1.0), with no sequence dependent score terms^20^. The steric radius of Val was used for that of the side chain pseudo atom. The ss_pair and rsigma score terms are modified so that only the strand residue pairs, specified in the blueprint, are favored in the simulations. Note that as for the interactions between helices only the steric repulsion (vdw term) is considered. The fragment assembly method was employed to build backbone structures^24^. Backbone fragment sets consisting of 1, 3, or 9 consecutive residue fragments, in which each fragment contains phi, psi, and omega torsion angle information, were prepared in advance from a nonredundant set of X-ray structures. In each Monte Carlo trial, a new conformation is generated by replacing the torsion angles (phi, psi, and omega) of a randomly selected frame consisting of 1, 3, or 9 consecutive residues with those of a randomly selected fragment compatible with the secondary structure and extended ABEGO type assigned in the blueprint. The number of Monte Carlo steps in one trajectory is 300 x (length of simulated chain) and the simulated temperature is 2.0. Different from our previous work^9^, the B region in the ABEGO torsion bins was further divided into the C, D, Y, and Z regions to sample backbone structures with more canonical structures for each loop type (Supplementary Fig.1). (Step 2) After building backbone structures with a target topology, for facilitating the β-sheet formation, the entire structures were minimized with the constraints making the Cα atoms of the neighboring strand residues in the blueprint being less than 5.5 Å, using the Rosetta full-atom FastRelax protocol^25^ with the score12 function with the upweighted hydrogen bonding and backbone torsion angle terms (hbond_sr_bb = 5.0, hbond_lr_bb = 3.0, and omega = 3.0). Val was used for the full-atom side chains for all residues except for those at the G region in the ABEGO Ramachandran map (Gly was used). After the minimization, the structures forming a designated β-sheet were used for carrying out the next step (here, the residues except for those at the both ends of the strands are required to be defined as strand residues by DSSP^23^). (Step 3) To make the end residues of each strand form hydrogen bondings, the loop–helix–loop and loop–helix motifs connecting the β-strands were rebuilt one by one from the N-terminus, using the CCD loop closure method implemented in the RosettaRemodel protocol^26^ with the Rosetta score function described above. After rebuilding these motifs, the structures are required to contain no helix kink and form the secondary structures and extended ABEGO torsions designated by the blueprint. In addition, the helices are required to interact with the β-sheet (see Calculation of buriedness of helix in Methods). Since the structures, in which the C-terminal helix tilts orthogonal against the β-strand direction and interacts with only the C-terminal edge residues of the β-strands, are observed, the constraint of the distance between the N-terminal residue at the first strand and the C-terminal residue at the last helix to be less than 15 Å was applied to avoid sampling such structures.

### Sequence design protocol

Sequence design was performed based on the protocol using the RosettaDesign approach^5^ with the extensions described in the paper^6^. We made an additional extension in this paper: polar amino acids were favored for the first and last residues of a β-strand to reduce the number of consecutive hydrophobic residues in β-strands as much as possible for preventing aggregation (Supplementary Fig. 3). This extension was not applied for the design of R2×3_BP1_A. In addition, the χ2 angle for the aromatic residues, F, Y, H,and W, was limited to the range from 70° to 110° frequently observed in nature^6^, but this extension was not applied for the design of R2×3_BP1_A and R3×3_BP1. Furthermore, except for the design of R2×3_BP1_A, internal β-strands were designed to have different hydrophobic residue patterns as much as possible using a variety of hydrophobic amino acids (AVILMF), seeking to prevent strand swapping from a standpoint of sequence design. For all the designs, we allowed the secondary structures and ABEGO torsions of the backbone structures being perturbed from the original ones as the result of the sequence design.

### Calculation of buriedness of helix

After rebuilding the loop–helix–loop and the loop–helix motifs, the buriedness of helix was evaluated to select the structures of which helices interact with the β-sheet. To this end, the accessible surface area of each helix residue represented by the coarse-grained model described above was calculated with a probe radius 2.0 Å using all the strand residues and the residues in the motif that were selected for rebuilding (Note that the other motifs were not included to exclude the “attractive” interaction of the rebuilding motif with the other motifs). We considered residues with the accessible surface area <40 Å^2^ as buried, and required at least one of the helix residues in each five consecutive residue window to be buried.

### Calculation of averaged backbone structure

Backbone structures generated from the above-described protocol were used for computing their averaged structure. First, the generated backbone structures (only mainchains) were superposed to a randomly selected backbone structure, and then the averaged xyz-coordinates were computed for the main-chain atoms. This procedure was performed again by superposing the backbone structures to the computed averaged coordinates, resulting in the averaged backbone structure. The averaged backbone structure was then idealized to have the bond lengths and bond angles close to the ideal values by using the Rosetta Idealization protocol with the upweighted score terms (hbond_sr_bb = 10.0, hbond_lr_bb = 10.0, and omega = 10.0).

### β-sheet formation probability

The β-sheet formation probability for each blueprint was calculated using a backbone ensemble generated by the above-described backbone building protocol. The probability was defined by the following formula: Σ (log *P*_*i*_) in which *P*_*i*_ is the formation probability of the *i*-th intra-β-sheet hydrogen bond in a backbone ensemble. To determine whether each hydrogen bond in a structure is formed or not, the Rosetta hydrogen bonding score (hbond_lr_bb) less than −0.01 was used.

### Packability between N- and C- terminal helices

The method for calculating the distance between the N- and C- terminal helices was described in Supplementary Fig. 8. The packability between the helices is defined as the log of the probability of the distance less than 11 Å in a backbone ensemble.

### Protein expression and purification

For all designed sequences except those for Pl2×3_BP, a Gly-Ser spacer was added between the C-terminus of the designed region and a 6xHis tag. The genes encoding the designed sequences except those for R3×3_BP2 and R3×3_BP3 were obtained from GenScript, which were cloned into plasmid pET29b for those for Pl2×3_BP, R2×3_BP1_A, R2×3_BP1_B, R3×3_BP1, and pET21b for those for R2×3_BP2. The genes for R3×3_BP2 were purchased from Gen9 and we cloned them into pET21b vector. The genes for R3×3_BP3 were obtained from FASMAC, which were cloned into pET21b vector. The designed proteins were expressed in *E. coli* BL21 Star (DE3) cells as uniformly (U-)^15^N-labeled proteins for all designs. The U-^15^N-labeled proteins were expressed by using MJ9 minimal media^27^, which have ^15^N ammonium sulfate as a sole nitrogen source and ^12^C glucose as a sole carbon source. The expressed proteins with a 6xHis tag were purified using a nickel affinity column and then dialyzed. We used PBS buffer, 137 mM NaCl, 2.7 mM KCl, 10 mM Na_2_HPO_4_, 1.8 mM KH_2_PO_4_, at pH 7.4, for all of the experiments other than NMR structure determination. The protein samples for CD measurements were then purified via gel filtration chromatography (AKTA pure 25 system with Superdex 75 Increase 10/300 GL column, GE Healthcare). The expression, solubility, and purity of the designed proteins were validated by SDS-PAGE and mass spectrometry (TSQ LC/MS, Thermo Scientific, was used for all designs except those of R3×3_BP3. For all designs of R3×3_BP3, Bruker Daltonics REFLEX III was employed).

### Circular dichroism (CD) spectroscopy

All CD data were collected in a 1 mm path length cuvette on a JASCO J-1500KS CD spectrometer. Far-UV CD spectra of designed proteins were measured from 260 to 200 nm at various temperatures from 30 up to 170 °C for 16–22 μΜ protein samples in PBS buffer (pH 7.4). The CD measurements at the temperature above 100 °C were made possible using HTC-572 unit, which can prevent protein samples from being boiled by raising the temperature under 1 MPa pressure. The protein concentrations were obtained from the absorbance at 280 nm^28^ detected by UV spectrophotometer (NanoDrop, Thermo Scientific). Thermal denaturation curves were measured at 222 nm at heating rate of 1 °C/min and the curves were fit with a sigmoidal function using nls function in R programming to obtain the temperature at the midpoint of the transition, *T*_*m*_. For R2×3_BP1_A5, the temperature range between 30 and 106 °C was regarded as a baseline of denatured state since the baseline is obscure. For R3×3_BP3_3, *T*_*m*_ was obtained without the fitting as the transition is linear.

### Size exclusion chromatography combined with multi-angle light scattering (SEC-MALS)

SEC-MALS measurements were performed by a miniDAWN TREOS static light scattering detector (Wyatt Technology) connected with a HPLC system (LC 1200 Series, Agilent Technologies). The volume 100 μl of 300–600 μΜ protein samples was injected into a Superdex 75 or Superdex 75 Increase 10/300 GL column (GE Healthcare) equilibrated with PBS buffer. The absorbance at 280 nm detected by the HPLC system was used for obtaining protein concentrations and scattered light intensity at 658 nm was detected at three different angles, 41.4°, 90.0°, and 138.6°. These data were analyzed by the ASTRA software (Wyatt Technology), in which a change in the refractive index with concentration, a *dn/dc* value, 0.185 ml/g, was used.

### 2D ^1^H-^15^N HSQC measurement

To confirm the core packing of designed proteins, we measured 2D ^1^H-^15^N HSQC spectra for all designs that were monomeric and had the αβ-protein CD spectrum. The spectra were collected for 0.2–1.5 mM protein samples in 90% ^1^H_2_O/10% ^2^H_2_O PBS buffer (pH 7.4) at 25 °C on a Varian INOVA 600 MHz spectrometer for the designs of Pl2×3_BP, R2×3_BP1_A, R2×3_BP1_B, R3×3_BP1, and R3×3_BP2, on a Bruker 800 MHz spectrometer for the designs of R2×3_BP4 and on a JEOL JNM-ECA 600 MHz spectrometer for the designs of R3×3_BP3, and were processed and analyzed using AutoProc/NMRpipe, Bruker TopSpin and JEOL Delta NMR software, respectively.

### Determination of solution structures by nuclear magnetic resonance (NMR) spectroscopy

The six designs were expressed and purified according to the standard largely-automated NESG protocol^29^. The designs were expressed in *E. coli* BL21 (DE3) pMGK cells as U-^15^N,5%^13^C-enriched proteins, and U-^15^N,U-^13^C-enriched proteins incubating MJ9 minimal media^27^. The U-^15^N, 5%^13^C-labeled proteins were used for stereo-specific assignments of methyl groups of valine and leucine^30^ and for residual dipolar coupling (RDC) measurements^31^. The expressed proteins were purified following an ÄKTAxpress™ (GE Healthcare) two-step protocol composed of IMAC (HisTrap HP column, GE Healthcare) and gel filtration chromatography (HiLoad 26/60 Superdex 75 column, GE Healthcare). The purified proteins were dissolved in 90% ^1^H_2_O/10% ^2^H_2_O buffer: 10 mM Tris-HCl, 100 mM NaCl, 5 mM DTT, 0.02% NaN_3_, at pH 7.5, for Pl2×3_BP_7; 20 mM MES, 200 mM NaCl, 10 mM DTT, 5 mM CaCl_2_, 0.02% NaN_3_, at pH 6.5, for R2×3_BP1_A5, R2×3_BP1_B9, R2×3_BP4_7, R3×3_BP1_9, and for R3×3_BP3_3. The expression level, solubility, and purity of the six proteins were confirmed by SDS-PAGE and MALDI-TOF mass spectrometry.

Solution NMR structure determination was performed without any knowledge of the design models. All NMR spectra for structure determination were acquired at 25 °C using cryogenic NMR probes. Triple resonance NMR data were collected on the Varian INOVA 600 MHz spectrometer or on a Bruker AVANCE 800 MHz spectrometer, while simultaneous 3D ^15^N/^13^C_aliphatic_/^13^C_aromatic_-edited NOESY^32^ (mixing time: 100 ms) and 3D ^13^C-edited aromatic NOESY (mixing time: 100 ms) spectra were measured on the Bruker AVANCE 800 MHz spectrometer. 2D constant-time ^1^H-^13^C HSQC spectra were acquired with 28 ms and 42 ms constant-time delays for the U-^15^N, 5%^13^C-enriched samples on the Varian INOVA 600 MHz spectrometer in order to obtain stereo-specific assignments of methyl groups of valine and leucine^30^. Backbone ^15^N-^1^H RDCs in two alignment media, PEG and phage, were acquired from J-modulated spectra^31^ for R2×3_BP1_B9. All of NMR data were processed using the program NMRPipe^33^ and analyzed using the program XEASY^34^. External DSS was used as a reference for spectra. Sequence-specific resonance assignments were determined using conventional triple-resonance NMR data, and analyzed automatically^35,36^ using the software AutoAssign^37^, followed by interactive validation and extension of side chain resonance assignments using XEASY^34^. Backbone dihedral angle constraints were derived from the chemical shifts using the program TALOS+^38^ or TALOSN^39^ for residues in well-defined secondary structure elements, and used for structure determination. Initial NOESY peak lists including expected intra-residue, sequential, and α-helical medium-range NOE peaks were created from the obtained assignments and then manually edited by visual inspection of the NOESY spectra. Subsequent manual peak picking was then performed to identify remaining, primarily long-range NOEs^36^. RDCs were used as orientational constraints for well-defined residues in the structure determination for R2×3 BP1_B9. The program CYANA^40,41^ was used to automatically assign NOEs and to calculate the structure. The 20 conformers with the lowest target function value were refined in explicit water^42^ using the program CNS^43^. RPF analysis of ASDP^44,45^ was used in parallel to guide the iterative cycles of noise/artifact peak removal, peak picking, and NOE assignments. The finally obtained structure coordinates were deposited in the Protein Data Bank. The structural statistics and global structure quality factors including Verify3D^46^, ProsaII^47^, PROCHECK^48^, and MolProbity^49^ raw and statistical *Z*-scores were computed using PDBSTAT^50^ and PSVS 1.5^51^. The global goodness-of-fit of the final structure ensemble with the NOESY peak list was computed using the RPF analysis program^52^.

### Reporting Summary

Further information on research design is available in the Nature Research Reporting Summary linked to this article.

## Supplementary information

Supplementary Information

Peer Review File

Reporting Summary

## Data Availability

The solution NMR structures have been deposited in the wwPDB as PDB 5GAJ [10.2210/pdb5GAJ/pdb] (Pl2x3_BP_7), 2L69 [10.2210/pdb2L69/pdb] (R2x3_BP1_A5), 2LCI [10.2210/pdb2LCI/pdb] (R2x3_BP1_B9), 6XEH [10.2210/pdb6XEH/pdb] (R2x3_BP4_7), 2L82 [10.2210/pdb2L82/pdb] (R3x3_BP1_9), and 7KBQ [10.2210/pdb7KBQ/pdb] (R3x3_BP3_3). Chemical shift, NOESY peak list, and raw free induction decay (fid) data were deposited in the Biological Magnetic Resonance Bank under the accession numbers 30000 [10.13018/BMR30000] (Pl2x3_BP_7), 17304 [10.13018/BMR17304] (R2x3_BP1_A5), 17613 [10.13018/BMR17613] (R2x3_BP1_B9), 30763 [10.13018/BMR30763] (R2x3_BP4_7), 17390 [10.13018/BMR17390] (R3x3_BP1_9), and 30802 [10.13018/BMR30802] (R3x3_BP3_3). The computational design models are available at https://github.com/kogalab21/global_bbstrain. The plasmids encoding the designed sequences are available from the authors upon request.

## Code availability

The code for calculating average structures and bend angles of β-sheets was implemented into Rosetta as ave_structs and ab_geom, respectively, at https://github.com/RosettaCommons/main/tree/koga/mydesign. Directions for obtaining a Rosetta license, downloading the Rosetta source code, and compiling the code are available on the RosettaCommons Web site (https://www.rosettacommons.org/software/license-anddownload).
