# Supplementary material for: Role of backbone strain in de novo design of complex α/β protein structures
Source: Nat Commun. 2021 Jun 24;12:3921. doi: 10.1038/s41467-021-24050-7 (PMC8225619; doi:10.1038/s41467-021-24050-7)
Supplement: Supplementary file 1 — Supplementary Information [file 41467_2021_24050_MOESM1_ESM.pdf]

Role of backbone strain in de novo design of complex α/β protein structures

Nobuyasu Koga[1,2,3,4,7*], Rie Koga[1,3,7], Gaohua Liu[5,7], Javier Castellanos[1], Gaetano T. Montelione[6*], and David Baker[1*]

[1]University of Washington, Department of Biochemistry and Howard Hughes Medical Institute, Seattle, Washington 98195, USA.
[2]Research Center of Integrative Molecular Systems, Institute for Molecular Science, National Institutes of Natural Sciences, Okazaki, Aichi 444-8585, Japan.
[3]Protein Design Group, Exploratory Research Center on Life and Living Systems (ExCELLS), National Institutes of Natural Sciences, Okazaki, Aichi 444-8585, Japan.
[4]SOKENDAI, The Graduate University for Advanced Studies, Shonan Village, Hayama, Kanagawa 240-0193, Japan.
[5]Nexomics Biosciences, 5 Crescent Ave, Rocky Hill, New Jersey 08553, USA.
[6]Department of Chemistry and Chemical Biology, and Center for Biotechnology and Interdisciplinary Sciences, Rensselaer Polytechnic Institute, Troy, New York 12180, USA.
[7]These authors contributed equally: Nobuyasu Koga, Rie Koga, Gaohua Liu.
[*]e-mail: nkoga@ims.ac.jp; dabaker@u.washington.edu; monteg3@rpi.edu

Contents

Supplementary Figure 1 | Extended ABEGO torsion bins and loop ABEGO patterns. 3

Supplementary Figure 2 | Part-by-part backbone building for the design target topologies. 4

Supplementary Figure 3 | The number of consecutive hydrophobic residues in β-strands. 5

Supplementary Figure 4 | Comparison between the design model and NMR structure for Pl2x3_BP_7. 6

Supplementary Figure 5 | Comparison between the design models and NMR structures for swapped Rossmann-fold designs. 7

Supplementary Figure 6 | Rosetta energy score comparison of the design models with the swapped NMR structures. 8

Supplementary Figure 7 | Part-by-part backbone building based on the NMR-structure-based blueprints. 9

Supplementary Figure 8 | Definition for the distance between the first and last helices. 10

Supplementary Figure 9 | Distribution of the helix distance for each backbone ensemble. 12

Supplementary Figure 10 | Definition for β-sheet curvature. 13

Supplementary Figure 11 | The packed helices generated by strongly bent β-sheets and distorted local backbone. 14

Supplementary Figure 12 | Agreement of the NMR structures with populated structures in the backbone ensembles. 15

Supplementary Figure 13 | Illustration of how the register shift generates bending of β-sheet. 16

Supplementary Figure 14 | Packing between terminal helices in the ensemble average structure for each blueprint. **17**

Supplementary Figure 15 | β-sheet hydrogen-bond formation probabilities in the structure ensemble for each blueprint. **18**

Supplementary Figure 16 | Rosetta energy score comparison of the design models with the unswapped NMR structures. **19**

Supplementary Figure 17 | Oligomerization state of designs for three folds by SEC-MALS. **20**

Supplementary Table 1 | Summary of experimental results for designed proteins **21**

Supplementary Table 2 | Summary of experimental results of 12 designs for Pl2x3_BP. **22**

Supplementary Table 3 | Summary of experimental results of 18 designs for R2x3_BP1_A. **23**

Supplementary Table 4 | Summary of experimental results of 13 designs for R2x3_BP1_B. **24**

Supplementary Table 5 | Summary of experimental results of 8 designs for R2x3_BP4. .... **25**

Supplementary Table 6 | Summary of experimental results of 12 designs for R3x3_BP1 .... **26**

Supplementary Table 7 | Summary of experimental results of 16 designs for R3x3_BP2 .... **27**

Supplementary Table 8 | Summary of experimental results of 10 designs for R3x3_BP3 .... **28**

Supplementary Table 9 | Summary of experimental results for representative designs in each design round for the three folds. **29**

Supplementary Table 10 | NMR and refinement statistics for protein structures*. **31**

Supplementary Table 11 | Designed sequences. **32**

SI References **39**

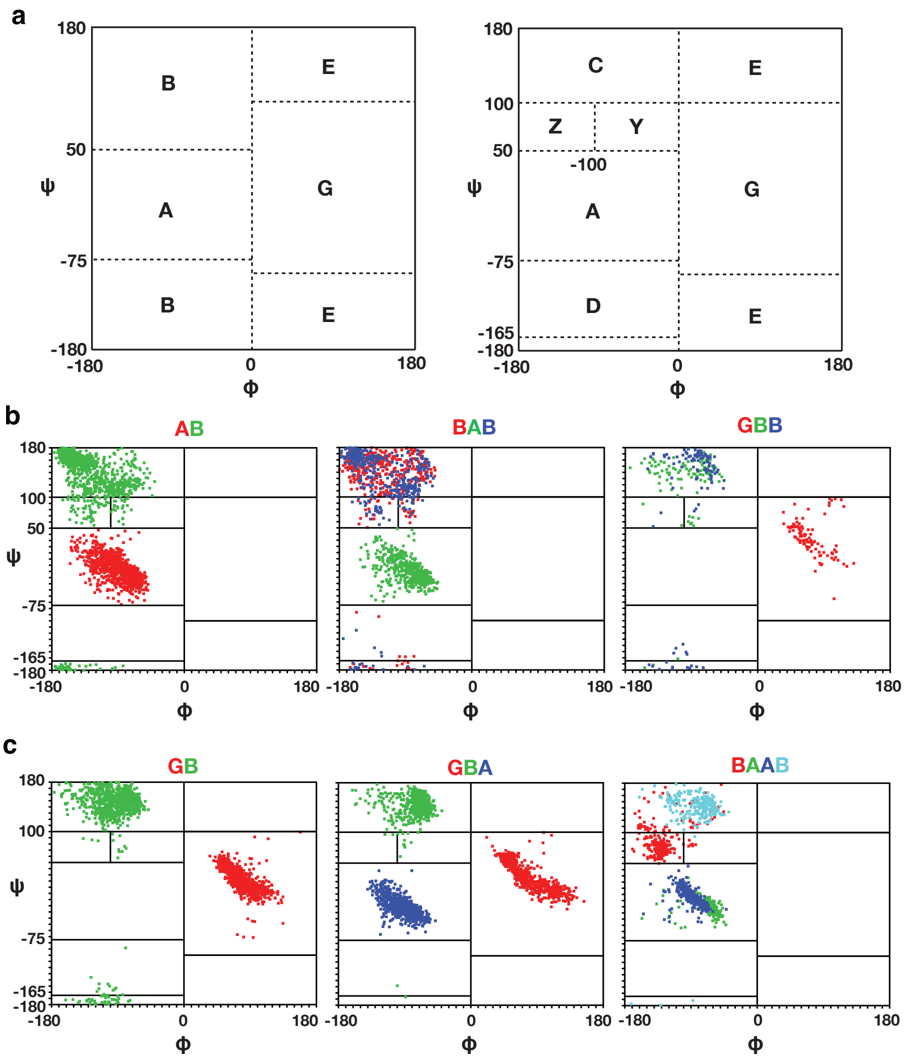

Supplementary Figure 1 | Extended ABEGO torsion bins and loop ABEGO patterns.

a, (left) The ABEGO torsion bins in the Ramachandran map described in the previous paper[S1]. (right) The extended ABEGO torsion bins. The B torsion was divided into the C and the other torsions, D, Y and Z, because residues with D, Y and Z torsions are rarely observed in nature due to the steric hindrance between the Cβ atom and mainchain atoms or between mainchain atoms[S2]. The Y and Z torsions are discriminated since the Z torsion is favored only for pre-proline residues despite the steric repulsion[S2]. **b**, The phi-psi distribution of the ABEGO loop types connecting β-strand to α-helix, AB, BAB and GBB loop types for naturally occurring proteins[S1]. **c**, The phi-psi distribution of the ABEGO loop types connecting α-helix to β-strand. In **b**, **c**, the color indicates the residue order: the first residue is red; the second, green; the third, blue; and the fourth, cyan. The D, Y and Z torsions are rarely observed, except for the Z torsion at the first B position in the BAAB loop type (this loop favors proline at the first A position[S1]).

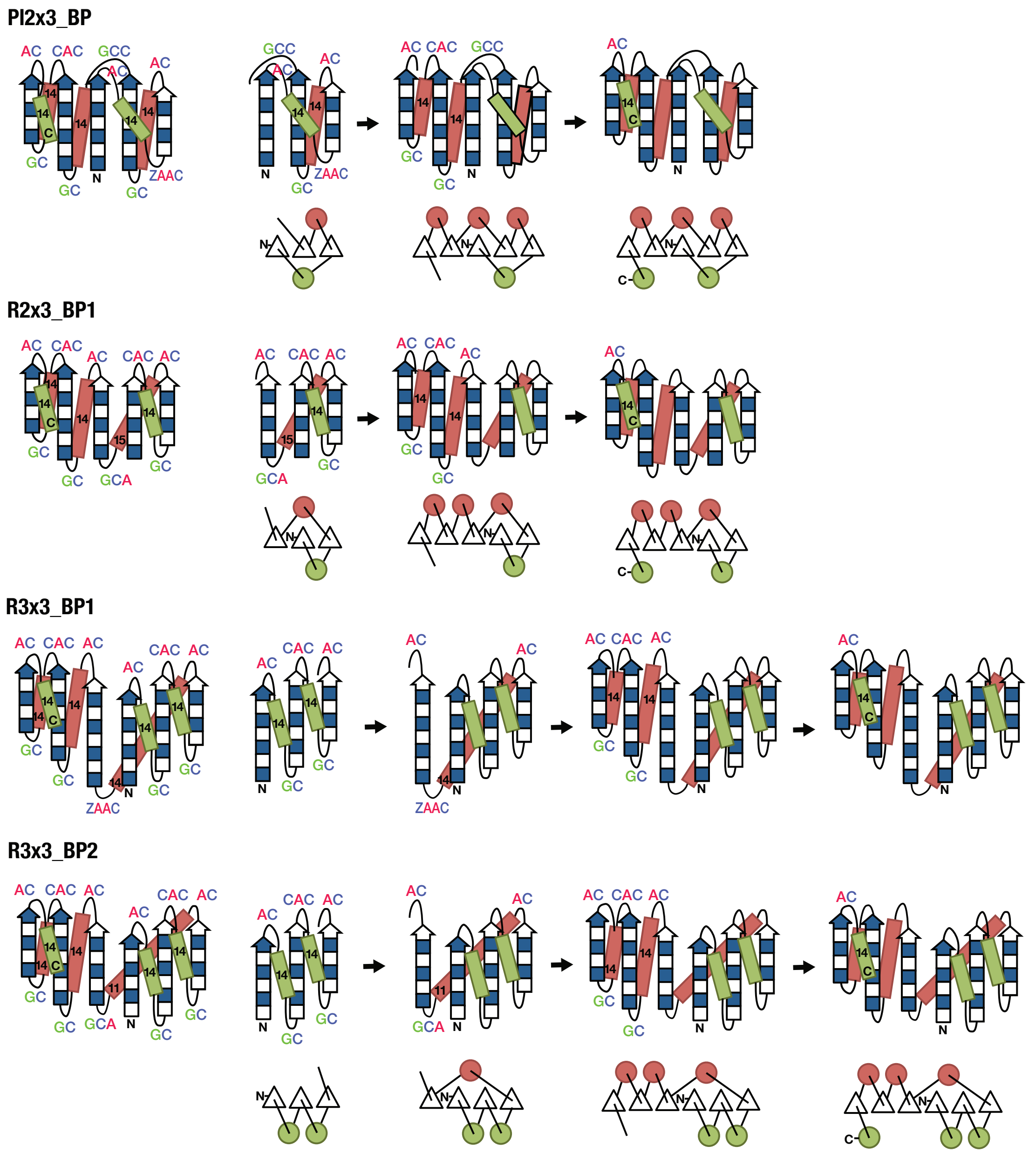

Supplementary Figure 2 | Part-by-part backbone building for the design target topologies.

Backbone structures were built part by part to increase the sampling efficiency. In the left, the full blueprints for Pl2x3_BP, R2x3_BP1, R3x3_BP1 and R3x3_BP2 same as Fig. 1 are shown, and at the following right, the steps of the part-by-part building are illustrated. The secondary structures and loops represented with their lengths or extended ABEGO loop patterns are newly built parts based on the structures built in the previous step.

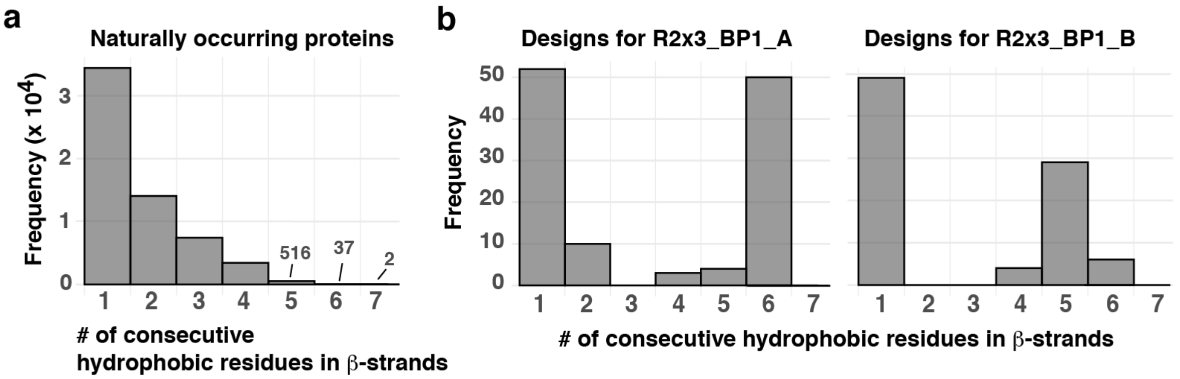

Supplementary Figure 3 | The number of consecutive hydrophobic residues in β-strands.

Distributions of the number of consecutive hydrophobic residues, Val, Ile, Leu, Met, Phe, Tyr and Trp, along the linear sequence in β-strands. **a**, The distribution for naturally occurring protein structures obtained from the PISCES server[S3] (6875 X-ray structures in the PDB with resolution ≤ 2.5 Å, R-factor ≤ 0.3, sequence lengths ≥ 40, and ≤ 25% sequence identity). **b**, The distribution for designed proteins for R2x3_BP1_A and R2x3_BP1_B.

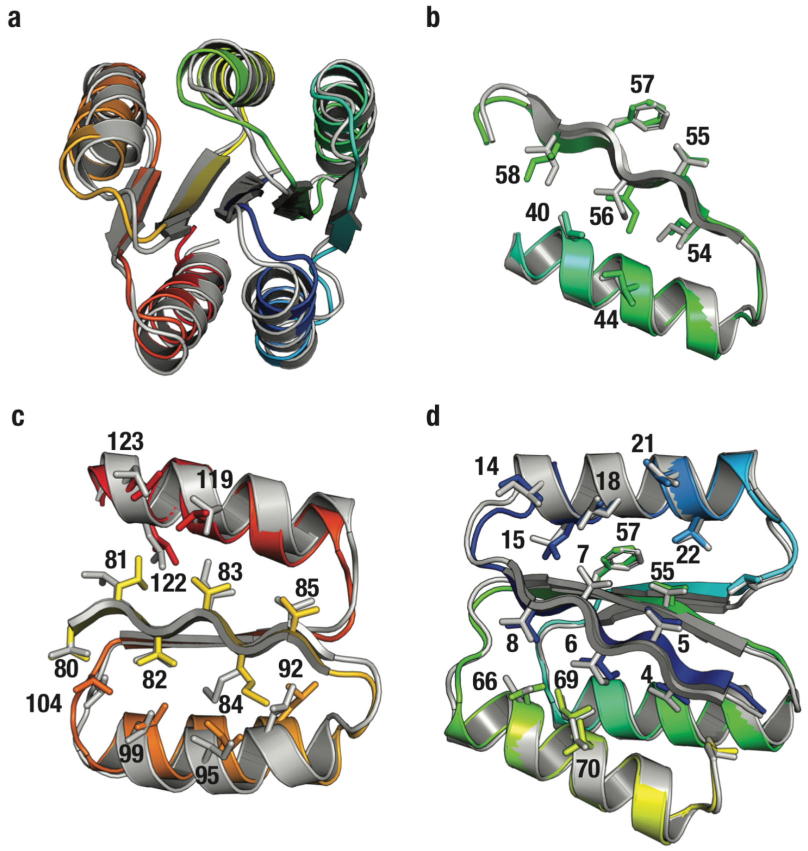

Supplementary Figure 4 | Comparison between the design model and NMR structure for Pl2x3_BP_7.

The design model (rainbow) and NMR structure (grey) for Pl2x3_BP_7 are superimposed (the Cα root mean square deviation is 1.1 Å). **a**, The backbone comparison. **b-d**, The comparison of core sidechain packing. The first model in the NMR structure (PDB: 5gaj) was used for the comparison.

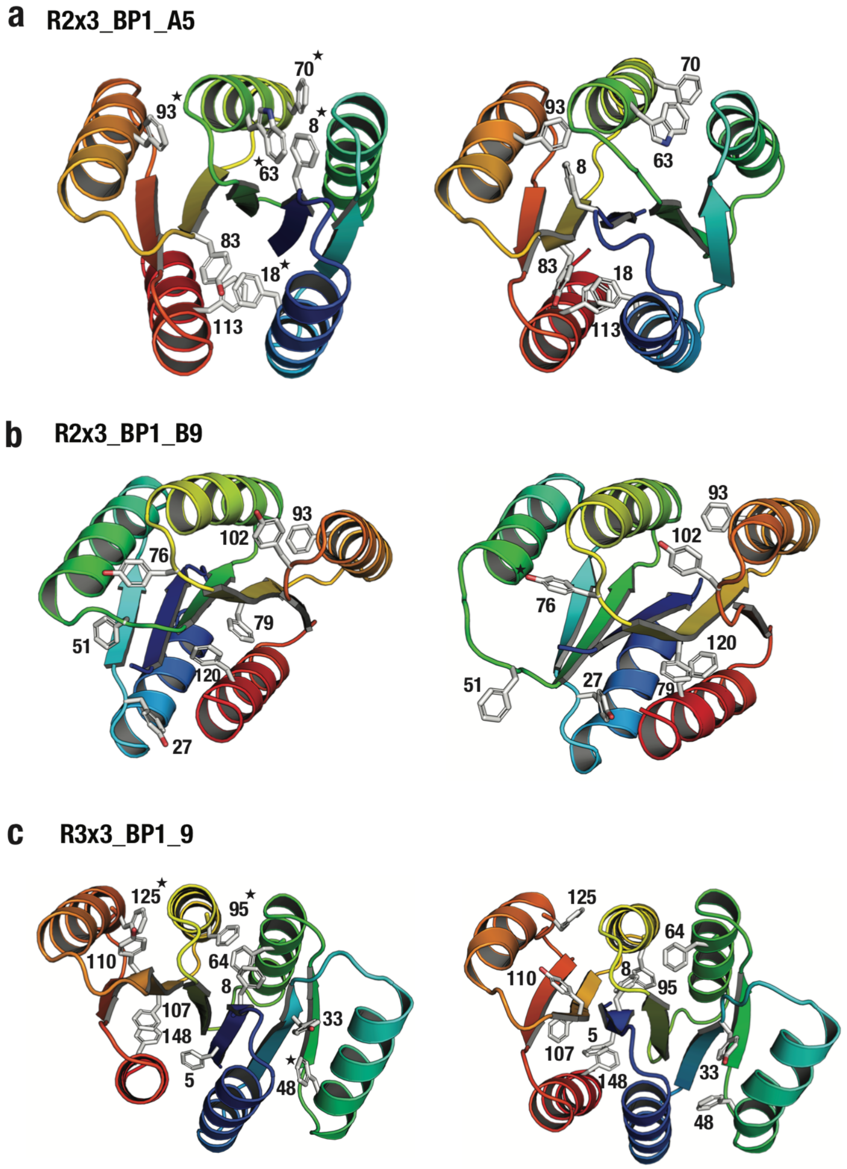

Supplementary Figure 5 | Comparison between the design models and NMR structures for swapped Rossmann-fold designs.

a, R2x3_BP1_A5, **b**, R2x3_BP1_B9, **c**, R3x3_BP1_9. Design models (left) and NMR structures (right). Aromatic residues are shown by stick to present the core packing difference between the design and NMR structures. The residues designed with the $\chi 2$ angle close to 0°, which is not frequently observed in nature, are indicated by a star.

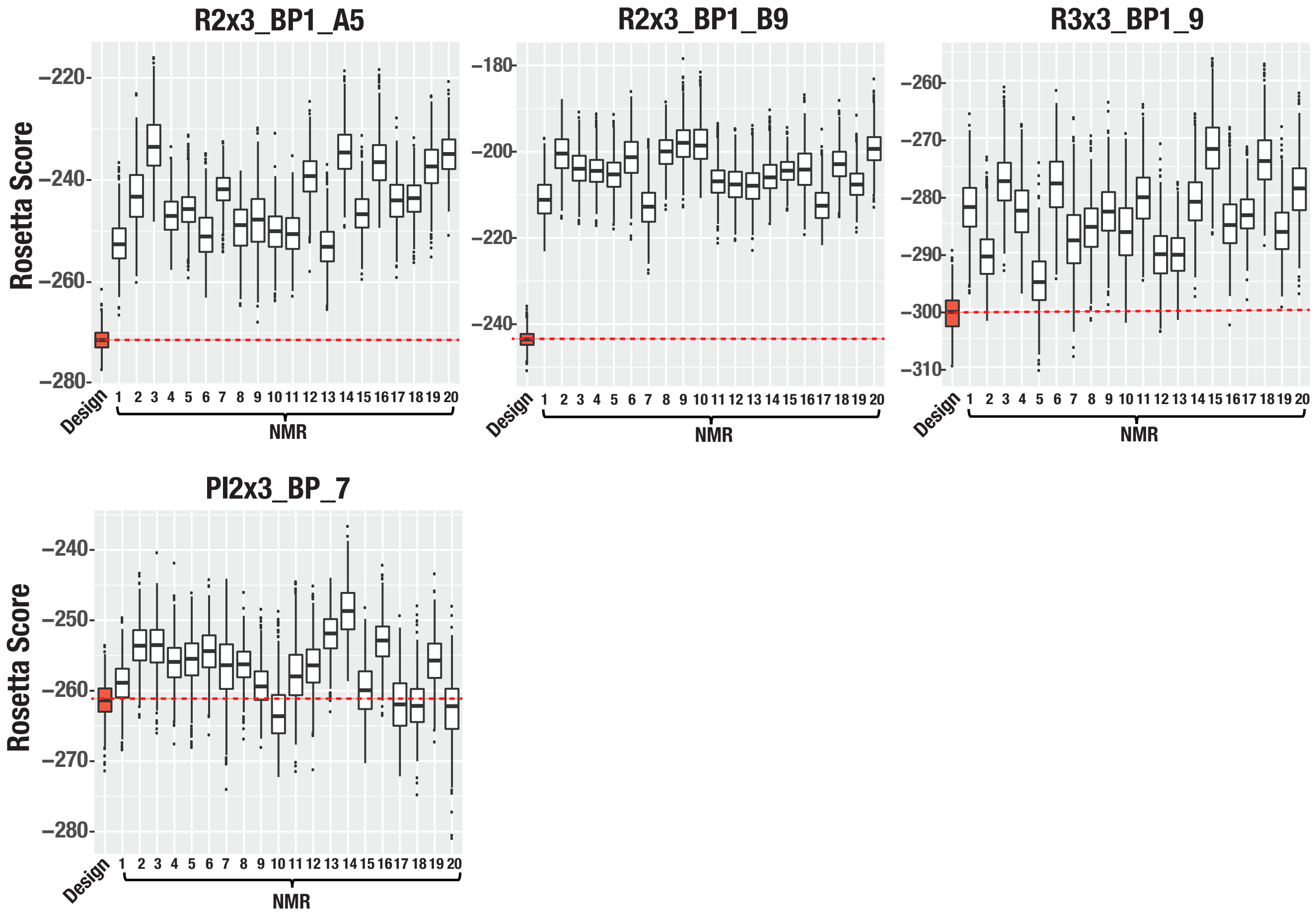

Supplementary Figure 6 | Rosetta energy score comparison of the design models with the swapped NMR structures.

(top) The Rosetta scores for the design models and their strand-swapped NMR structures (20 NMR models) were plotted by boxplots. (bottom) The Rosetta scores for the design, Pl2x3_BP_7 (Ploop2x3 fold), were also shown as a reference. These scores were computed after the structure minimization by the Rosetta FastRelax protocol[S4] with the score-weight, beta_nov15[S5]. The thick line in box is median; the upper and lower lines of box are upper and lower quartile ($Q_{3/4}$ and $Q_{1/4}$), respectively; the dots are outliers, more than $Q_{3/4}$ + 1.5 x ($Q_{3/4}$ - $Q_{1/4}$) or less than $Q_{1/4}$ - 1.5 x ($Q_{3/4}$ - $Q_{1/4}$); and the upper whisker is maximum value but less than outliers, the lower whisker is minimum value but more than outliers. n = 1000 independent structure minimization trajectories for score computations.

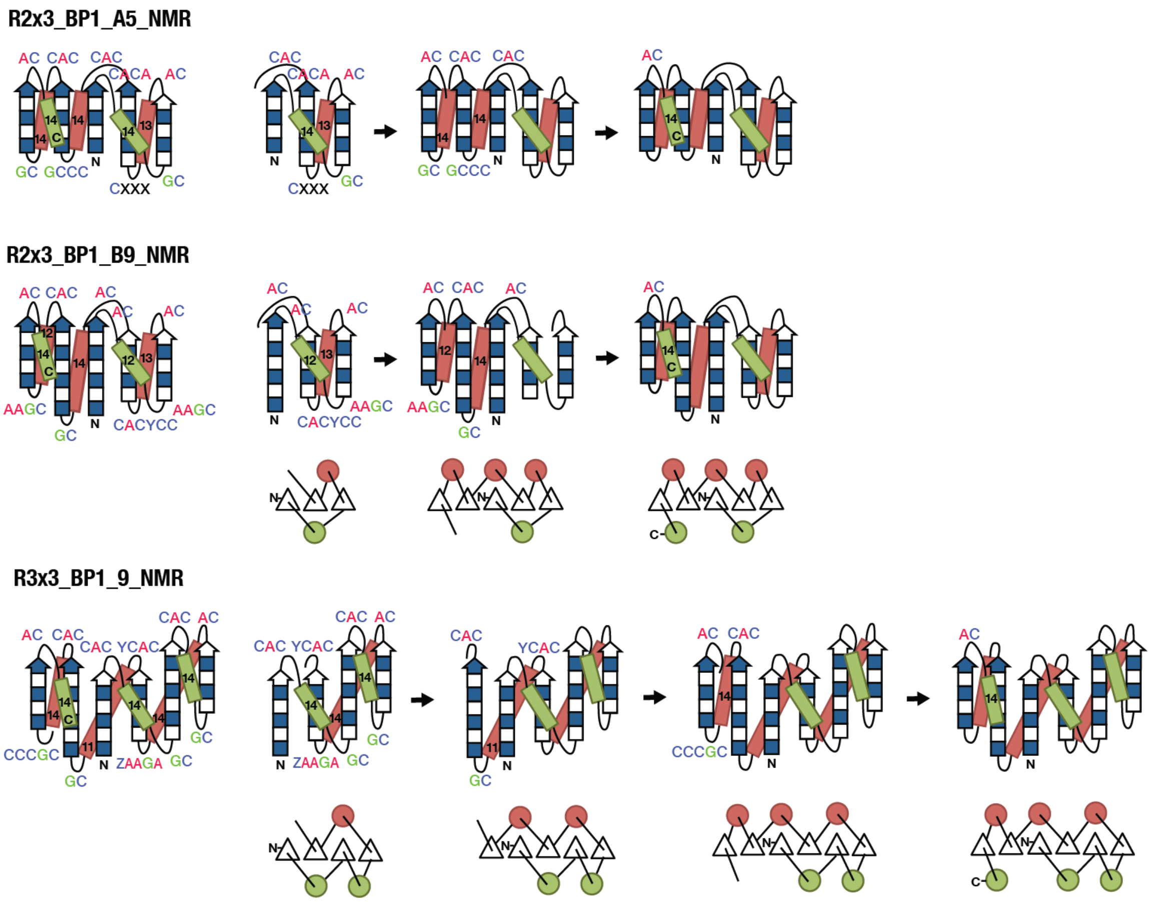

Supplementary Figure 7 | Part-by-part backbone building based on the NMR-structure-based blueprints.

Backbone structures were built part by part to increase the sampling efficiency. In the left, the full blueprints for R2x3_BP1_A5_NMR, R2x3_BP1_B9_NMR, and R3x3_BP1_9_NMR are shown. At the following right, the steps of the part-by-part building are illustrated. The torsion pattern for each residue in the loops was determined by looking up the consensus of the extended ABEGO torsion in the NMR structures. The residues with X have no consensus and therefore do not have the ABEGO torsion constraint during the backbone building. The secondary structures and loops with their lengths or the extended ABEGO loop patterns are newly built parts based on the structures built in the previous step.

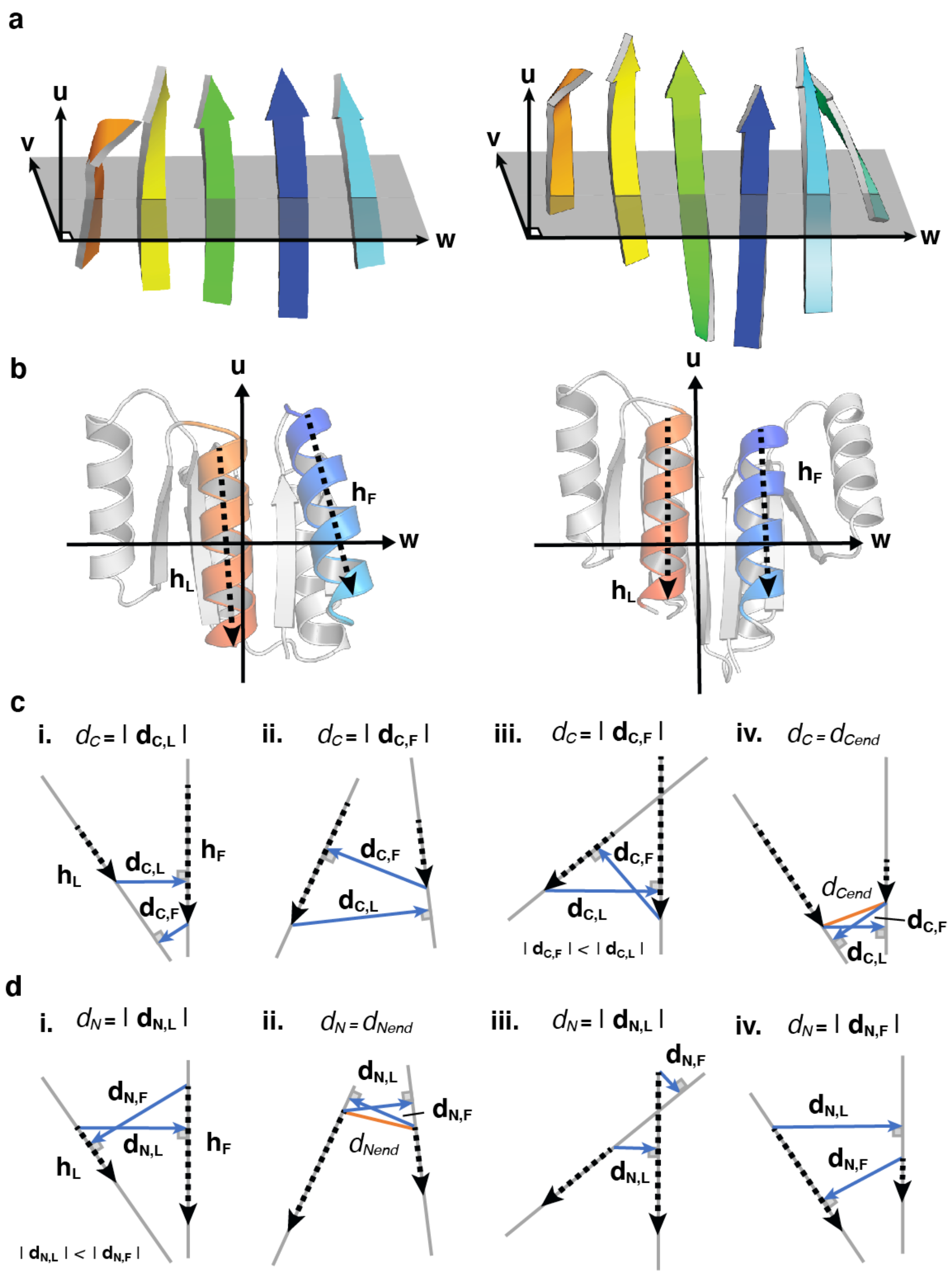

Supplementary Figure 8 | Definition for the distance between the first and last helices.

a, The three orthogonal vectors, **u**, **v**, and **w**, characterizing the β-sheets for five-stranded (left) and six-stranded (right) topologies. The vectors were defined by the eigenvectors calculated by the principal component analysis of the five strand vectors, each of which is obtained by averaging unit vectors from the N to the C atoms of the strand residues except for the first and the last, belonging to the β-strand. **b**, The distance between the first and the last helices for five-stranded (left) and six-stranded (right) topologies, d, was identified on the **uw**-plane defined in **a**. First, a helix vector was defined as a vector from the N-terminal center averaged over N-terminal

eleven atoms (the backbone N, Cα, and C atoms of the first three residues and the N and Cα atoms of the fourth residue) to the C-terminal center averaged over C-terminal eleven atoms (the backbone N, Cα, and C atoms of the last three residues and the Cα and C atoms of the fourth residue from the last). To compute d, the helix vectors of the first and last helices are projected onto the **uw**-plane, resulting in the projected helix vectors $\mathbf{h}_F$ and $\mathbf{h}_L$**.** Then, d was defined as the average distance of d_N (the distance between the N-terminals of the helix vectors) and d_C (the distance between the C-terminals), which were obtained using $\mathbf{h}_F$ and $\mathbf{h}_L$ in the followings. **c**, The d_C distance was identified with the four patterns, i-iv, depending on the relative placement of the projected $\mathbf{h}_F$ and $\mathbf{h}_L$ helix vectors. We considered the vectors $\mathbf{d}_{C,F}$ and $\mathbf{d}_{C,L}$ starting from the terminal of $\mathbf{h}_F$ and $\mathbf{h}_L$, each of which is orthogonal to $\mathbf{h}_L$ and $\mathbf{h}_F$. Depending on whether or not the $\mathbf{d}_{C,F}$ and $\mathbf{d}_{C,L}$ vectors intersect with $\mathbf{h}_L$ and $\mathbf{h}_F$, there are the following four patterns: (i)(ii) either $\mathbf{d}_{C,F}$ or $\mathbf{d}_{C,L}$ vectors has the intersection; (iii) both have the intersections; and (iv) no intersection. For (i) and (ii), d_C is the length of the vector that has the intersection. For (iii), d_C is the length of the vector that is shorter than the other. For (iv), d_C is the length between the C-terminals of the projected helix vectors. **d**, The d_N distance was defined in the same fashion as d_C.

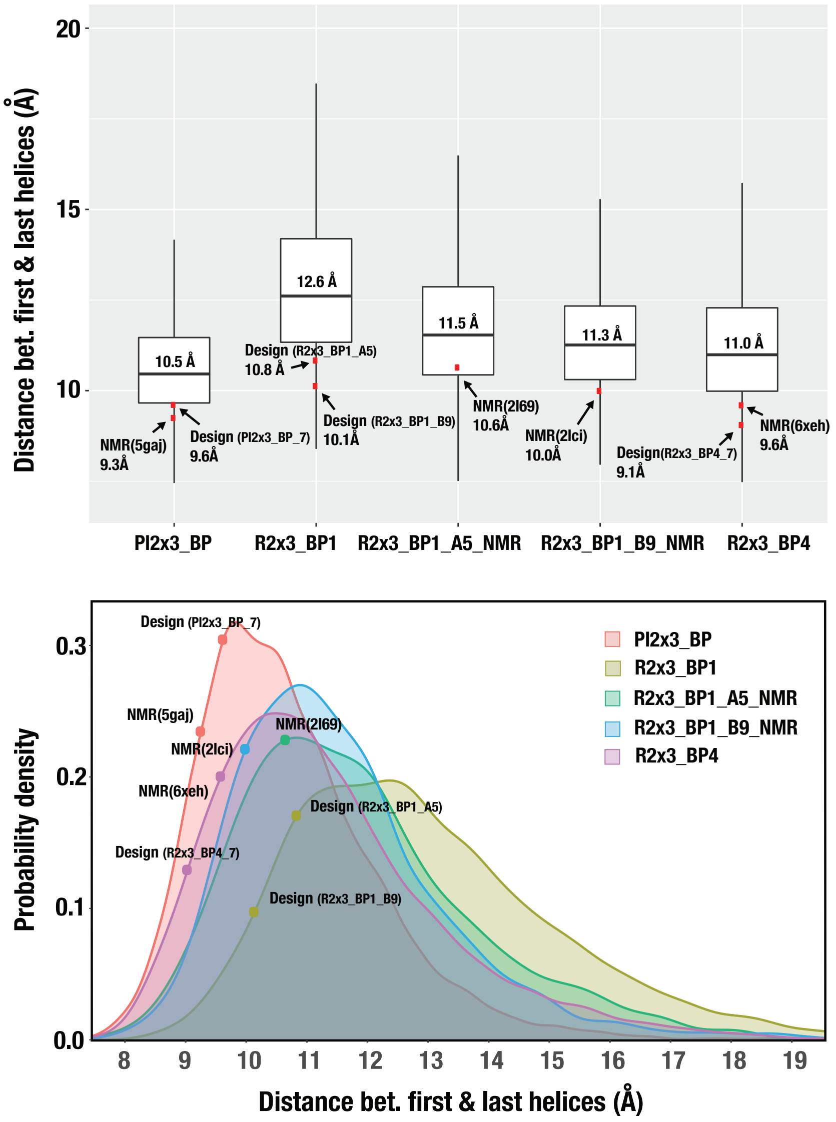

Supplementary Figure 9 | Distribution of the helix distance for each backbone ensemble.

(top) The helix distance distributions for the backbone ensembles generated from Pl2x3_BP, R2x3_BP1, R2x3_BP1_A5_NMR, R2x3_BP1_B9_NMR, and R2x3_BP4 were plotted by boxplots (the ensembles respectively contain n = 9719, 6176, 1427, 1011, 8058, independently generated backbone structures). The thick line in box is median; the upper and lower lines of box are upper and lower quartile ($Q_{3/4}$ and $Q_{1/4}$), respectively; the outliers, defined as more than $Q_{3/4}$ + 1.5 x ($Q_{3/4}$ - $Q_{1/4}$) or less than $Q_{1/4}$ - 1.5 x ($Q_{3/4}$ - $Q_{1/4}$), are not plotted; and the upper whisker is maximum value but less than outliers, the lower whisker is minimum value but more than outliers. The red points indicate the distances for the design or the averaged NMR structures. (bottom) The same distributions as the top ones were plotted by probability density distributions.

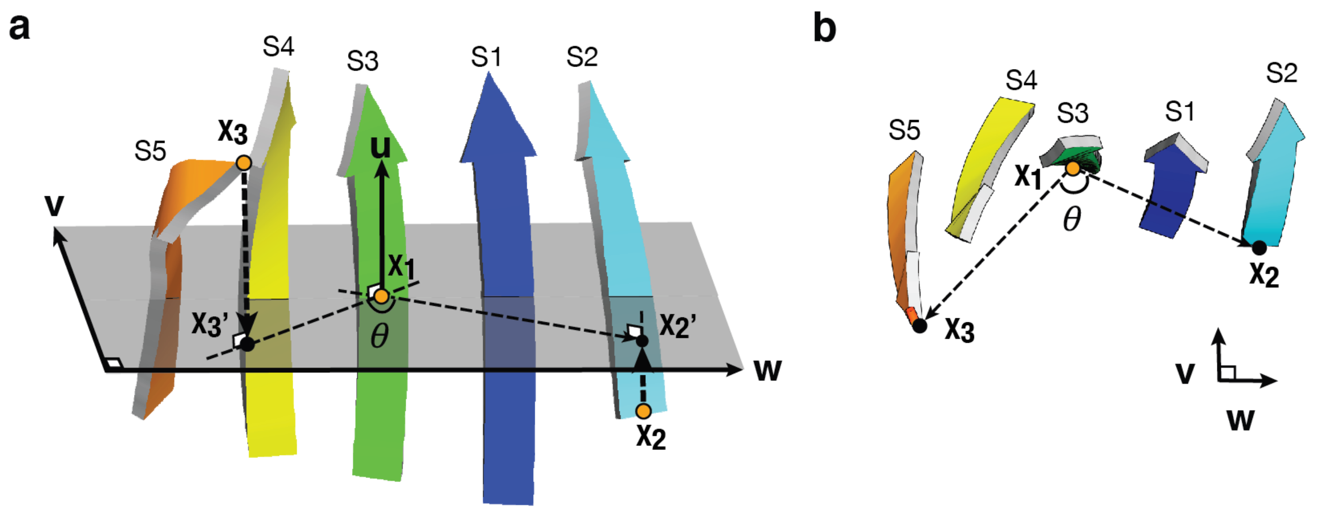

Supplementary Figure 10 | Definition for β-sheet curvature.

a, The definition for β-sheet curvature, θ, is illustrated for Rossmann2x3 fold (the same definition was used for Ploop2x3 fold). This fold has the β-sheet with the strand ordering, 2-1-3-4-5, in which each number represents the order of β-strand along the linear chain (the symbols starting from S in the figures represent the orders). First, the β-sheet was characterized by its center coordinates, $\mathbf{x}_1$, and its three orthogonal directional vectors, **u**, **v**, and **w**. The center was obtained by averaging the N (backbone amide nitrogen) and the C (backbone carbonyl carbon) atom coordinates of the 3rd strand (S3) residues except for the first and last residues. The three orthogonal directional vectors are same as those described in Supplementary Figure 8. Then, the Cα coordinates of the first residue in the β-strand at the right edge of the β-sheet (i.e., the 2nd β-strand), $\mathbf{x}_2$, and the Cα coordinates of the last residue of the β-strand at the left edge of the β-sheet (i.e., the 5th β-strand), $\mathbf{x}_3$, were projected onto the plane perpendicular to the vector **u** through $\mathbf{x}_1$, resulting in $\mathbf{x}_2$' and $\mathbf{x}_3$'. Finally, the β-sheet curvature, θ, was computed by the angle between the vectors $\mathbf{x}_{1,2'}$ and $\mathbf{x}_{1,3'}$. **b**, The β-sheet curvature for Rossmann2x3 fold was viewed from the positive side of the **u** vector.

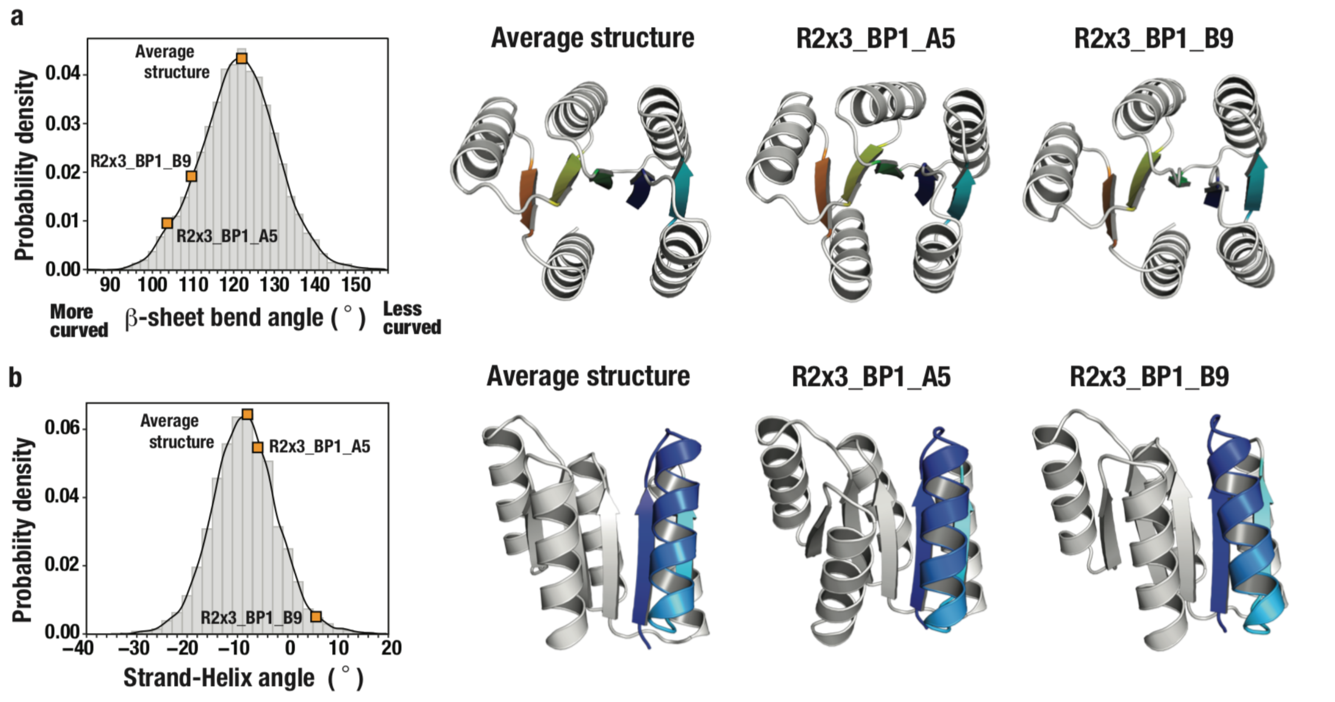

Supplementary Figure 11 | The packed helices generated by strongly bent β-sheets and distorted local backbone.

a, (left) The distribution of the β-sheet bend angle for the backbone ensemble generated from the R2x3_BP1 blueprint was plotted with the angles for the ensemble average structure and the designed structures, R2x3_BP1_A5 and R2x3_BP1_B9. (right) The ensemble average structure and the designed structures in the same view as Supplementary Fig. 10b. The β-sheet of the R2x3_BP1_A5 structure is strongly bent, and that of the R2x3_BP1_B9 structure is also bent, which makes the first and last helices close together. **b**, (left) The distribution of the strand-helix angle for the first β-α unit colored in blue was plotted with the ensemble average structure and the designed structures, R2x3_BP1_A5 and R2x3_BP1_B9. The strand-helix angle was defined as the dihedral angle between the strand vector, the strand-helix vector, and the helix vector of the β-α unit: the strand vector was defined as the vector from the midpoint of the N and C atoms of the first strand residue to that of the last strand residue; the strand-helix vector, from the midpoint of the N and C atoms of the last strand residue to the average of the first 11 backbone heavy atoms of the helix; and the helix vector is the same as defined in Supplementary Fig. 8. (right) The ensemble average structure and the designed structures. The backbone structure of the first β-α unit in the R2x3_BP1_B9 structure is strongly distorted, which makes the first and last helices close together.

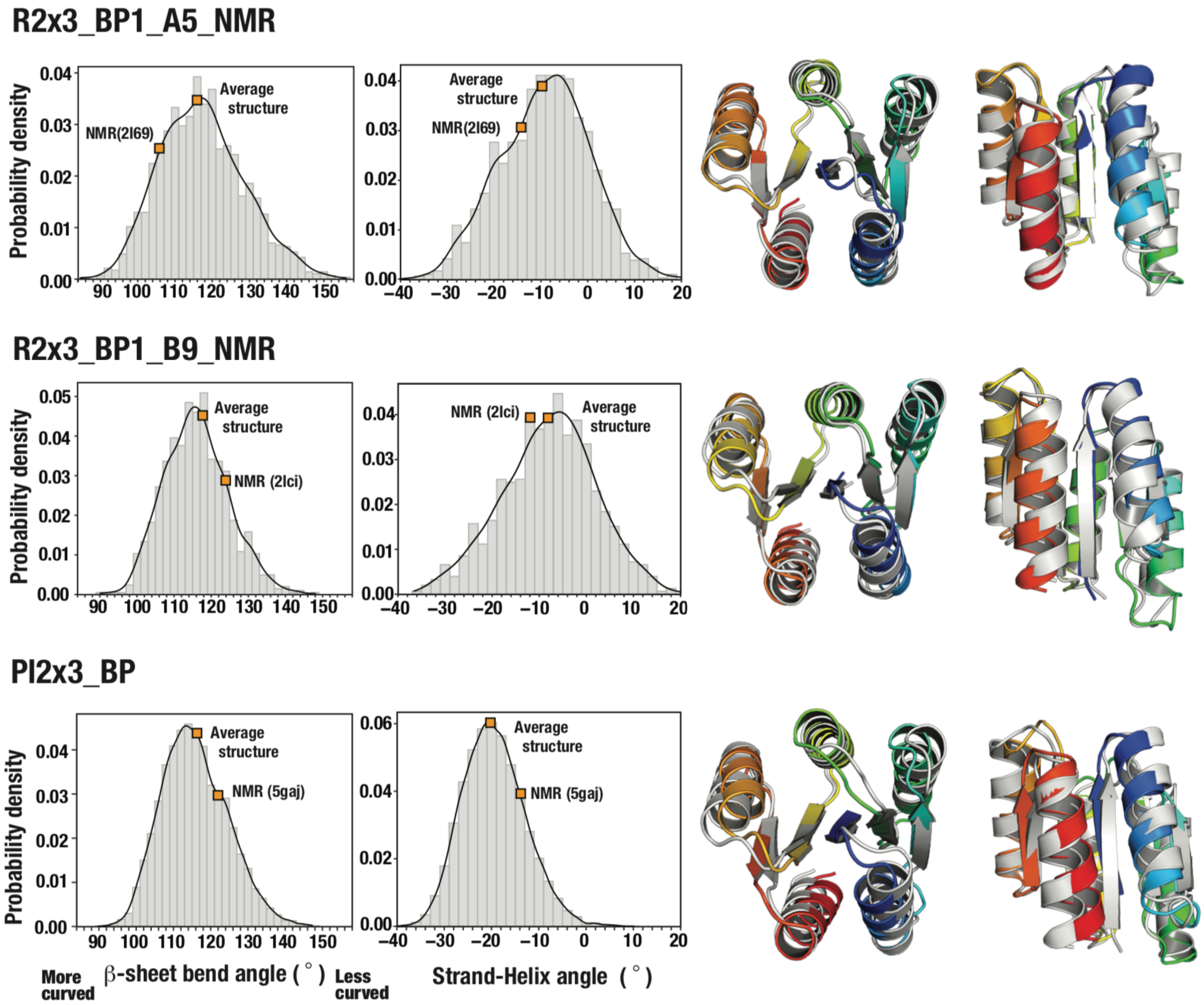

Supplementary Figure 12 | Agreement of the NMR structures with populated structures in the backbone ensembles.

(left) The distributions of the β-sheet bend angle and the strand-helix angle for the first β-α unit, as in Supplementary Fig. 11, for the backbone ensemble generated from the R2x3_BP1_A5_NMR, R2x3_BP1_B9_NMR, and Pl2x3_BP blueprints, were plotted with the angles for their ensemble average structures and the averaged NMR structures. (right) Superpositions of the averaged NMR structures (rainbow) with the backbone ensemble average structures (gray).

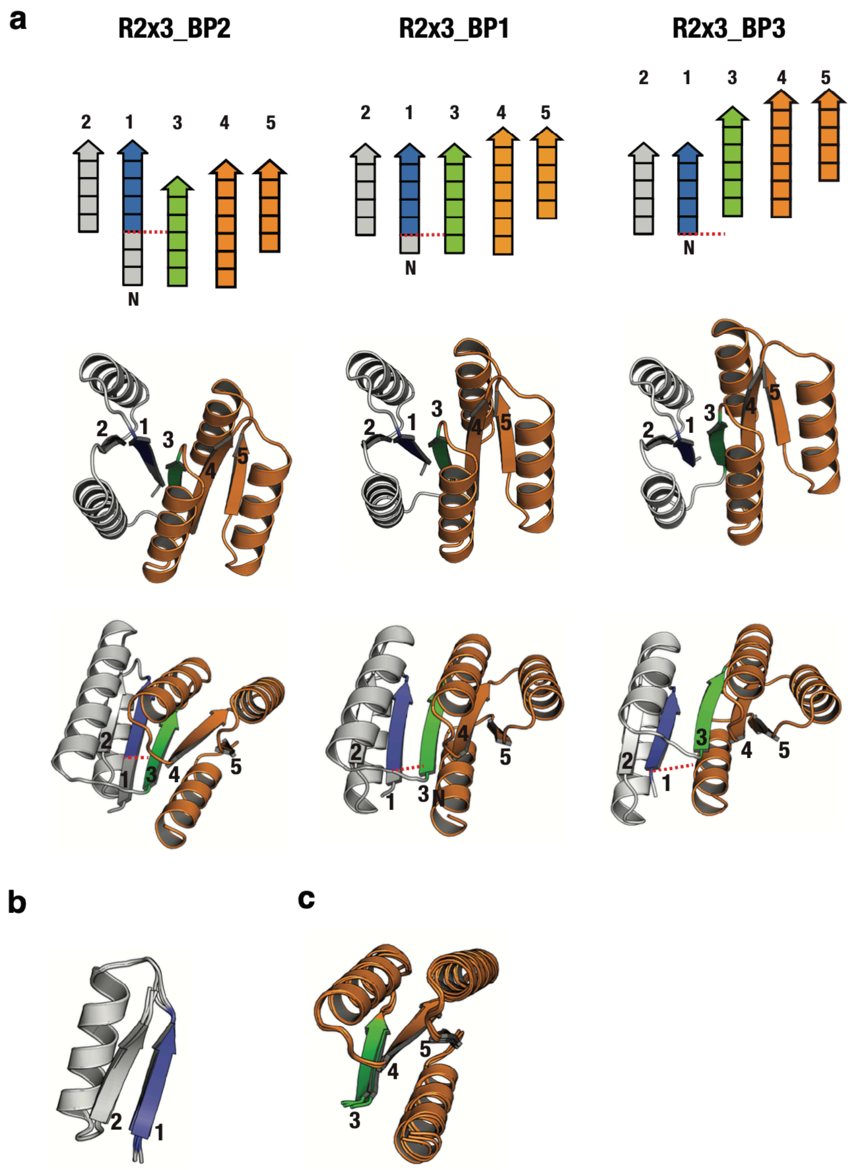

Supplementary Figure 13 | Illustration of how the register shift generates bending of β-sheet.

a, (top) The blueprints, R2x3_BP2, R2x3_BP1, and R2x3_BP3, with different registries between the first (blue) and third (green) β-strands. The red dotted line indicates the registry corresponding to the first strand residue position in the R2x3_BP3 blueprint. (middle and bottom) The average structures of the backbone ensembles generated from the blueprints. The number represents the β-stand order. **b**, Superpositions of the N-terminal half domains of the R2x3_BP1, R2x3_BP2, and R2x3_BP3 average backbone structures. **c**, Superpositions of the C-terminal half domains of the R2x3_BP1, R2x3_BP2, and R2x3_BP3 average backbone structures. The registry dependent rigid-body packing orientation of the C-terminal half (green and orange region) relative to the N-terminal half (grey and blue region) generates different β-sheet bending. The intra twisting of each strand is independent of this registry, and hence does not contribute to the curvature, which is different from the bending of anti-parallel β-sheets, in which the intra twisting of strands plays a role for the entire β-sheet bending[S6].

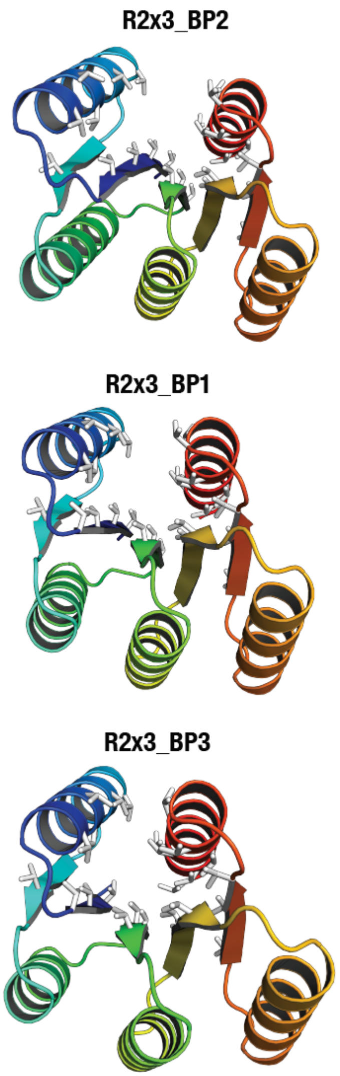

Supplementary Figure 14 | Packing between terminal helices in the ensemble average structure for each blueprint.

The ensemble average structures generated from three blueprints with a flatter β-sheet to more curved ones are shown. Valine sidechains were used for presenting the packing difference depending on the blueprints.

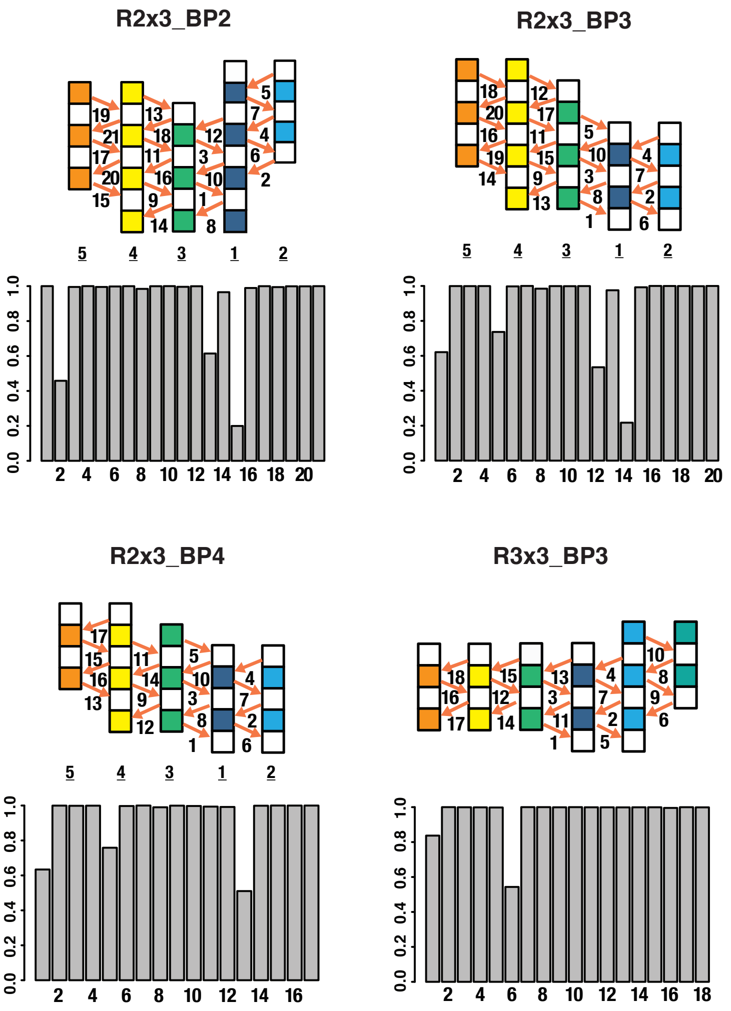

Supplementary Figure 15 | β-sheet hydrogen-bond formation probabilities in the structure ensemble for each blueprint.

(top) Schematic diagrams of hydrogen bonds formed in the parallel β-sheet. (bottom) The probability for each hydrogen bond being formed in the generated structure ensemble. (The numbering in the bar graph corresponds to that indicating the hydrogen bonds in the β-sheet schematics).

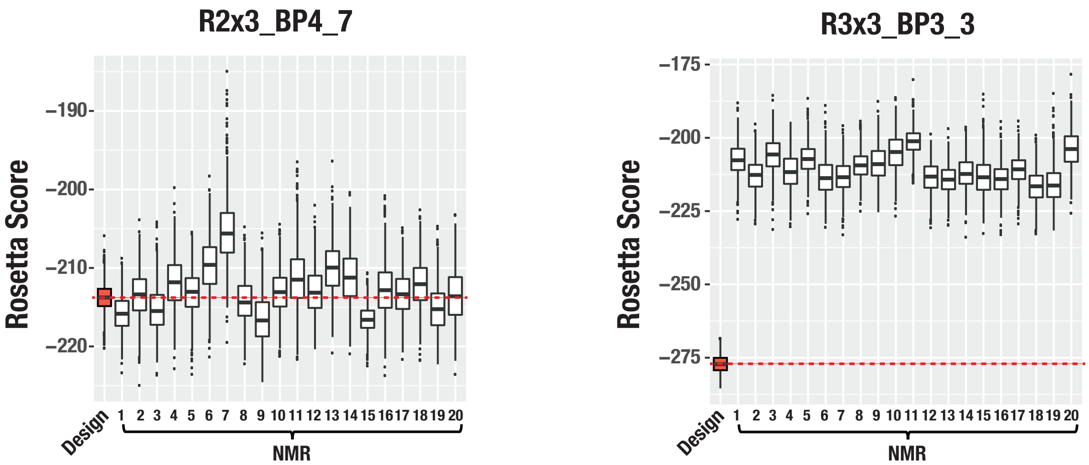

Supplementary Figure 16 | Rosetta energy score comparison of the design models with the unswapped NMR structures.

(top) The Rosetta scores for the design models and their unswapped NMR structures (20 NMR models) were plotted by boxplots. These scores were computed after the structure minimization by the Rosetta FastRelax protocol[S4] with the score-weight, beta_nov15[S5]. The thick line in box is median; the upper and lower lines of box are upper and lower quartile ($Q_{3/4}$ and $Q_{1/4}$), respectively; the dots are outliers, more than $Q_{3/4} + 1.5 \times (Q_{3/4} - Q_{1/4})$ or less than $Q_{1/4} - 1.5 \times (Q_{3/4} - Q_{1/4})$; and the upper whisker is maximum value but less than outliers, the lower whisker is minimum value but more than outliers. n = 1000 independent structure minimization trajectories for score computations.

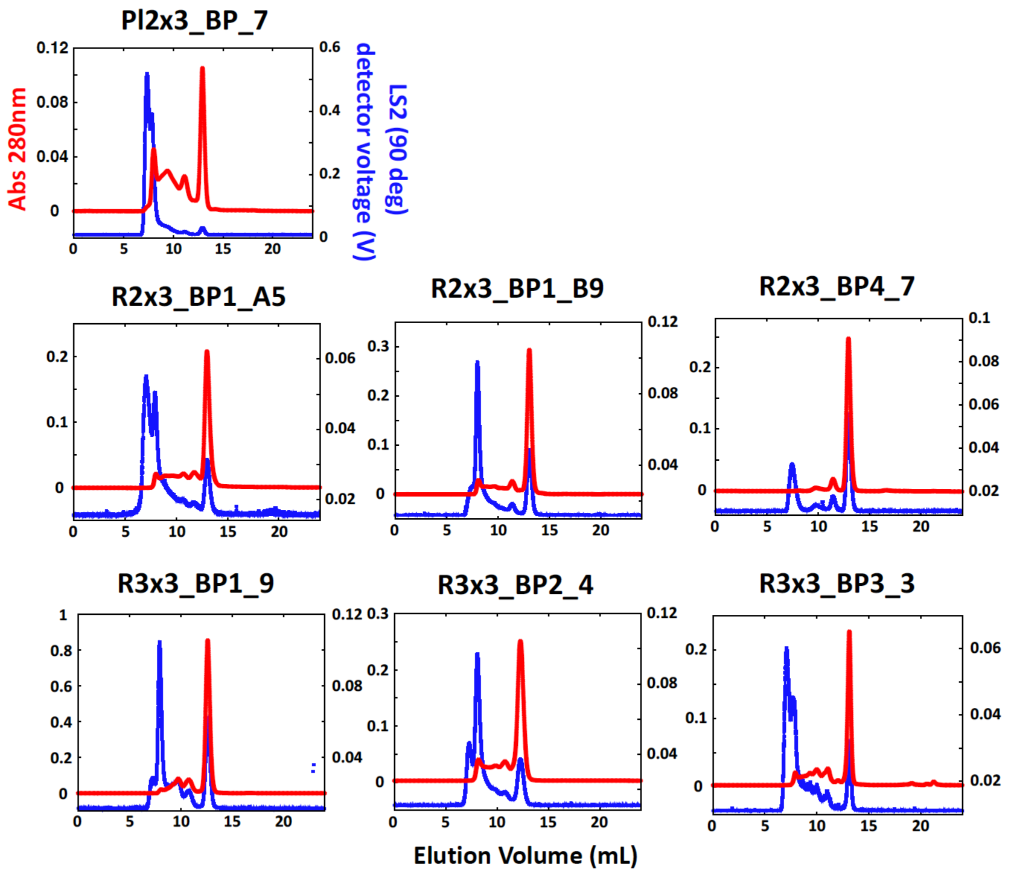

	theoretical MW (kDa)	estimated MW (kDa)
Pl2x3_BP_7	15.8	16.4
R2x3_BP1_A5	15.8	17.8
R2x3_BP1_B9	16.2	13.9
R2x3_BP4_7	14.6	15.1
R3x3_BP1_9	19.7	18.8
R3x3_BP2_4	18.2	18.2
R3x3_BP3_3	16.1	15.1

Supplementary Figure 17 | Oligomerization state of designs for three folds by SEC-MALS.

The volume 100 μl of 300-600 μM protein samples was injected into a Superdex 75 10/300 GL column equilibrated with PBS buffer (pH 7.4). The absorbance at 280 nm (red line) and the light scattering data of LS2 (90 degree) at 658 nm (blue line) were collected along the elution volume, which were analyzed to give the molecular weight (MW) of the main peak. The estimated MW by the analysis is presented in the third column in the table. The theoretical MW of monomer calculated from the design sequence is shown in the second column.

	#designs tested	Expressed[1]	Soluble[1]	αβ-protein CD spectrum (25 °C)	Stable[2] ($T_m \geq 95$ °C)	Monomeric[3]	Well resolved NMR[4]	Success (rate %)
**Pl2x3_BP**	12	11	5	5	5	2	1	1 (8)
**R2x3**								
**BP1_A**	18	18	15	14	14	7	1	
**BP1_B**	13	13	11	10	10	5	3	
**BP2**	8	8	8	7	6	6	5	5 (63)
**R3x3**								
**BP1**	12	12	12	12	12	9	1	
**BP2**	16	16	16	15	15	11	0	
**BP3**	10	10	5	5	5	4	2	2 (20)

Supplementary Table 1 | Summary of experimental results for designed proteins.

The second column shows the number of designs experimentally tested for the fold in the leftmost column. The subsequent columns give the number of designs that satisfy each experimental characterization, which was performed sequentially from the left to the right. The successful designs are defined as those that satisfy all criteria and are expected to fold into correct fold. The details of the results are shown in Supplementary Tables 2-8.

[1] Expression and solubility were assessed by SDS-PAGE and mass spectrometry.

[2] Stability was measured by thermal denaturation; T_m is the transition midpoint temperature.

[3] SEC-MALS was used to determine oligomerization state. The number of designs in which the main peak of the absorbance at 280 nm corresponds to the monomeric state was counted.

[4] ^{1}H-^{15}N HSQC spectra were collected.

	Expressed	Soluble	αβ-protein CD spectrum (25 °C)	T_m (°C)	Monomeric	Well-resolved HSQC
**1**	Y	N				
**2**	Y	Y	Y	> 95	N §	
**3**	Y	N				
**4**	Y	Y	Y	>> 95	N §	
**5**	Y	N				
**6**	Y	Y	Y	> 95	N	
**7**	Y	Y	Y	139	Y	Y
**8**	Y	N				
**9**	Y	Y	Y	>> 95	Y	N
**10**	Y	N				
**11**	Y	N				
**12**	N					

Supplementary Table 2 | Summary of experimental results of 12 designs for Pl2x3_BP.

Each row corresponds to the results for each design. The columns give the results for each experimental characterization, of which the details are described in Supplementary Table 1. Each characterization was performed sequentially from the left to the right; well-behaved designs at a characterization (Y) are then evaluated by the next one and not well-behaved designs (N) stop being evaluated.

§ The main peak of the absorbance at 280 nm was dimeric state.

	Expressed	Soluble	αβ-protein CD spectrum (25 °C)	T_m (°C)	Monomeric	Well-resolved HSQC
**1**	Y	Y	Y	>> 95	N	
**2**	Y	Y	Y	>> 95	Y	N
**3**	Y	Y	Y	>> 95	Y	N
**4**	Y	Y	Y	>> 95	Y	N
**5**	Y	Y	Y	141	Y	Y
**6**	Y	Y	Y	>> 95	N	
**7**	Y	Y	Y	>> 95	Y	N
**8**	Y	Y	Y	>> 95	Y	N
**9**	Y	Y	Y	>> 95	Y	N
**10**	Y	Y	Y	> 95	N	
**11**	Y	Y	Y	>> 95	N	
**12**	Y	Y	Y	>> 95	N	
**13**	Y	Y	Y	> 95	N	
**14**	Y	Y	Y	>> 95	N	
**15**	Y	N				
**16**	Y	Y	N			
**17**	Y	N				
**18**	Y	N				

Supplementary Table 3 | Summary of experimental results of 18 designs for R2x3_BP1_A.

The summary was given in the same way as the Supplementary Table 2.

	Expressed	Soluble	αβ-protein CD spectrum (25 °C)	T_m (°C)	Monomeric	Well-resolved HSQC
**1**	Y	Y	Y	>> 95	Y	N
**2**	Y	N				
**3**	Y	N				
**4**	Y	Y	Y	> 95	N	
**5**	Y	Y	Y	>> 95	Y	Y
**6**	Y	Y	Y	>> 95	Y	N
**7**	Y	Y	Y	>> 95	Y	Y
**8**	Y	Y	N			
**9**	Y	Y	Y	120	Y	Y
**10**	Y	Y	Y	>> 95	N	
**11**	Y	Y	Y	> 95	N	
**12**	Y	Y	Y	>> 95	N	
**13**	Y	Y	Y	>> 95	N	

Supplementary Table 4 | Summary of experimental results of 13 designs for R2x3_BP1_B.

The summary was given in the same way as the Supplementary Table 2.

	Expressed	Soluble	αβ-protein CD spectrum (25 °C)	T_m (°C)	Monomeric	Well-resolved HSQC
**1**	Y	Y	Y	>> 95	Y	Y
**2**	Y	Y	Y	>> 95	Y	Y
**3**	Y	Y	N			
**4**	Y	Y	Y	≈ 95	Y	Y
**5**	Y	Y	Y	74	Y	N
**6**	Y	Y	Y	>> 95	Y	Y
**7**	Y	Y	Y	96	Y	Y
**8**	Y	Y	Y	>> 95	N	

Supplementary Table 5 | Summary of experimental results of 8 designs for R2x3_BP4.

The summary was given in the same way as the Supplementary Table 2.

	Expressed	Soluble	αβ-protein CD spectrum (25 °C)	T_m (°C)	Monomeric	Well-resolved HSQC
**1**	Y	Y	Y	>> 95	Y	N
**2**	Y	Y	Y	>> 95	Y	N
**3**	Y	Y	Y	>> 95	Y	N
**4**	Y	Y	Y	>> 95	Y	N
**5**	Y	Y	Y	> 95	N	
**6**	Y	Y	Y	> 95	N	
**7**	Y	Y	Y	>> 95	Y	N
**8**	Y	Y	Y	>> 95	Y	N
**9**	Y	Y	Y	121	Y	Y
**10**	Y	Y	Y	> 95	Y	N
**11**	Y	Y	Y	>> 95	Y	N
**12**	Y	Y	Y	>> 95	N	

Supplementary Table 6 | Summary of experimental results of 12 designs for R3x3_BP1.

The summary was given in the same way as the Supplementary Table 2.

	Expressed	Soluble	αβ-protein CD spectrum (25 °C)	T_m (°C)	Monomeric	Well-resolved HSQC
**1**	Y	Y	N			
**2**	Y	Y	Y	> 95	Y	N
**3**	Y	Y	Y	>> 95	Y	N
**4**	Y	Y	Y	135	Y	N
**5**	Y	Y	Y	> 95	N	
**6**	Y	Y	Y	> 95	N	
**7**	Y	Y	Y	> 95	Y	N
**8**	Y	Y	Y	> 95	Y	N
**9**	Y	Y	Y	>> 95	Y	N
**10**	Y	Y	Y	>> 95	Y	N
**11**	Y	Y	Y	>> 95	N	
**12**	Y	Y	Y	>> 95	Y	N
**13**	Y	Y	Y	>> 95	Y	N
**14**	Y	Y	Y	>> 95	Y	N
**15**	Y	Y	Y	>> 95	N	
**16**	Y	Y	Y	>> 95	Y	N

Supplementary Table 7 | Summary of experimental results of 16 designs for R3x3_BP2.

The summary was given in the same way as the Supplementary Table 2.

	Expressed	Soluble	αβ-protein CD spectrum (25 °C)	T_m (°C)	Monomeric	Well-resolved HSQC
**1**	Y	N				
**2**	Y	N				
**3**	Y	Y	Y*	126	Y	Y
**4**	Y	Y	Y*	> 95	N	
**5**	Y	N				
**6**	Y	N				
**7**	Y	Y	Y*	> 95	Y	N
**8**	Y	Y	Y*	> 95	Y	Y
**9**	Y	N				
**10**	Y	Y	Y*	> 95	Y	N

Supplementary Table 8 | Summary of experimental results of 10 designs for R3x3_BP3.

The summary was given in the same way as the Supplementary Table 2.

* The CD spectrum was characteristic of αβ-proteins, but looked partially unfolded.

	RMSD between design and NMR (Å)		T_m (°C)
	**Cα atoms**	**Heavy atoms**	
**Pl2x3_BP_7**	1.1	2.1	139
**R2x3_BP1_A5**	Ploop2x3 fold		141
**R2x3_BP1_B9**			120
**R2x3_BP4_7**	1.8	2.6	96
**R3x3_BP1_9**	Ploop3x3 fold		121
**R3x3_BP2_4**	molten globule		135
**R3x3_BP3_3**	3.4	4.3	126

Supplementary Table 9 | Summary of experimental results for representative designs in each design round for the three folds.

The second and third columns show the average RMSD between the design model and the 20 NMR structures with the same correct fold using Cα atoms and heavy atoms respectively. The computationally designed region corresponds to the region from N=2 Gly to N=126 Val in the NMR structures (PDB code: 5gaj) for Pl2x3_BP_7, N=2 GLY to N=115 Gly in 6xeh for R2x3_BP4_7, and N=2 Arg to N=127 Ile in 7kbq for R3x3_BP3_3. These regions were used for RMSD calculations. The last column shows the temperature at the midpoint of the transition, T_m.

Design ID	Pl2x3_ BP_7	R2x3_ BP1_A5	R2x3_ BP1_B9	R2x3_ BP4_7	R3x3_ BP1_9	R3x3_ BP3_3
PDB ID	**5gaj**	**2l69**	**2lci**	**6xeh**	**2l82**	**7kbq**
BMRB ID	**30000**	**17304**	**17613**	**30763**	**17390**	**30802**
NESG ID	**OR258**	**OR28**	**OR36**	**OR386**	**OR32**	**OR689**
**NMR distance and dihedral restraints**						
Distance restraints						
Total NOE	3697	2776	3566	3989	5010	3128
Intra-residue	867	761	964	852	1137	901
Inter-residue						
Sequential (|i-j| = 1)	877	619	746	892	1148	825
Medium-range (|i-j| ≤ 4)	846	537	776	938	1041	717
Long-range (|i-j| ≥ 5)	1107	859	1080	1307	1684	685
Hydrogen bonds	68	88	58	80	82	86
Total dihedral angle restraints						
phi	106	100	98	88	120	97
psi	106	100	98	88	120	97
Total RDCs						
Q(%, alignment media 1§)			19.4			
Q(%, alignment media 2§)			23.9			
**Structure statistics**						
Violations						
Distance restraints¶ (Å)						
mean	0.001	0.000	0.001	0.001	0.001	0.001
rmsd	0.008	0.005	0.008	0.008	0.007	0.014
sd	0.008	0.005	0.008	0.008	0.007	0.014
Dihedral angle restraints (°)						
mean	0.132	0.025	0.148	0.070	0.067	0.360
rmsd	0.676	0.158	0.61	0.398	0.358	1.13
sd	0.663	0.158	0.614	0.392	0.352	1.07
Max. distance restraint violation (Å)	0.29	0.26	0.31	0.43	0.29	0.88
Max. dihedral angle restraint violation (°)	9.3	2.4	7.1	5.8	4.7	11.60
Average pairwise r.m.s.d.** (Å)						
Heavy	1.05±0.08	1.23±0.09	0.99±0.07	0.86±0.06	1.01±0.07	1.15±0.10
Backbone	0.56±0.09	0.66±0.09	0.57±0.09	0.42±0.06	0.48±0.05	0.71±0.12
RPF Scores						
Recall	0.975	0.98	0.986	0.976	0.981	0.925
Precision	0.974	0.949	0.969	0.976	0.972	0.929
F-measure	0.974	0.964	0.977	0.796	0.976	0.927
DP-scores	0.89	0.842	0.891	0.913	0.903	0.688†
Structure Quality Factors - overall statistics scores (raw/Z-scores⌘)						
Procheck G-factor (phi / psi only)**	0.14/0.87	0.15/0.90	0.15/0.901	0.14/0.87	0.02/0.39	-0.17/-0.35
Procheck G-factor (all dihedral angles)**	0.06/0.35	0.08/0.47	0.07/0.41	0.07/0.41	-0.05/-0.30	-0.27/-1.60
Verify3D	0.31/-2.41	0.25/-3.37	0.40/-0.96	0.32/-2.25	0.32/-2.25	0.16/-4.82
ProsaII (-ve)	1.51/3.56	1.20/2.27	0.86/0.87	1.40/3.10	1.40/3.10	0.89/0.99
MolProbity clashscore	9.94/-0.18	8.54/0.06	13.6/-0.81	9.67/-0.13	12.41/-0.6	9.48/-0.10
Ramachandran Plot Summary from Richardson Lab's Molprobity**						
Most favored regions (%)	98.2	98.9	99.1	99.4	98	97.4
Allowed regions (%)	1.7	1.1	0.9	0.6	2	2.6
Disallowed regions (%)	0.0	0.0	0.0	0.0	0.0	0.0

Supplementary Table 10 | NMR and refinement statistics for protein structures*.

* Analyzed for the 20 lowest energy refined structures of each design for the three folds by using PDBSTAT and PSVS 1.5[S7, 8].

§ PEG and phage were used as alignment media 1 and 2.

¶ Calculated by using sum over r^{-6}.

** Calculated among 20 refined structures for well-defined residues that have sum of phi and psi order parameters[S9] S(phi)+S(psi)>1.8[S7]. The well-defined residues of Pl2x3_BP_7: 3-60, 62-110, 112-128; R2x3_BP1_A5: 3-8, 11-47, 52-75, 77-124; R2x3_BP1_B9: 3-47, 53-128; R2x3_BP4_7: 4-114; R3x3_BP1_9: 3-127, 130-153; R3x3_BP3_3: 3-8, 18-74, 76-127. The represented structure (the medoid) is the model that has average lowest RMSD to the other models: Pl2x3_BP_7: #1; R2x3_BP1_A5: #2; R2x3_BP1_B9: #5; R2x3_BP4_7: #6; R3x3_BP1_9: #2; R3x3_BP3_3: #20.

† The relatively low DP score of this structure is attributable to extra NOESY peaks due to alternative states (lowering the recall score) and missing NOESY peaks due to exchange broadening (lowering the precision score). These dynamics also result in less complete convergence in the coordinates for the N and C terminal helices compared with the other NMR structures.

⌘ With respect to mean and standard deviation for a set of 252 X-ray structures with sequence lengths < 500, resolution ≤ 1.80 Å, R-factor ≤ 0.25 and R-free ≤ 0.28; a positive value indicates a 'better' score.

Supplementary Table 11 | Designed sequences.

Computationally designed sequences are shown in uppercase and residues added to allow expression, purification, and the spacer between the designed sequence and the N-terminal Met or the C-terminal His-tag are shown in lowercase.

Pl2x3_BP_1	mGTVVILVSRNDNILDRLVEEILKRDPNIVTTRVDNSDKVKDEIEKLLRKGRPVVVVITGVTASDIKDIVRELKKRGVNVLVIYVNTDDDKLKKMWEELQKDGVRVRVTDNENQAIKEMLRLLDELlehhhhhh
Pl2x3_BP_2	mGTVVLVVSNNDNDLDDLLKKVKEEDPNVRVTVTDNKEKVKEQAKRLLKKGRPVVIFITGVTAQKIKEIVEEMKKQGVNVVVIILDTDEEELRRIWRELQEEGVTVRVTDNLEEAKRRLVKVLREKlehhhhhh
Pl2x3_BP_3	mGTVVILLSNNDNILEKLVEELKKQDPNVVTTRVDNADKVSRDIKKILEEGYPVVVVVSGLTSSDIRKIVEDLKKSGVNVLVIIVDKDENRLRQIYRELQEEGVRVRTTDNREQAIREMLRLFDELlehhhhhh
Pl2x3_BP_4	mGTIIIVVSNNERVLEELLKEVLKQDPNVVTTRVDNRQKVEDVMEKAKQKGRPVVIFIRGATASVIRDIVEKAQKSGVNVVVIILDKDDEKLKRIWKDLQKKGVDVRVTDNEDTAKDVLKDVMDKKlehhhhhh
Pl2x3_BP_5	mGTVVIVVSDDENILEQLLKEVRKSDPNVVTTRVDDKEKVKKVIEDARKKGRPVVIFIRGATREMIRDIVKKAQEEGVRVLVIIVDKDENKLRKIYEQLREEGVDVRVTDNKEEAIKRLIEFLKKLlehhhhhh
Pl2x3_BP_6	mGTVVIVVSRDENILDELIRMVIEKDPNVVTTRVDDKNKVKEYIEEAIKRGRPVVIFIRGATRDVVRDIVEKLKKEGVRVLVIIVDQDDDELKKIYEQLKKEGVDVRVTDNRDEAIKLLLDLLEKGlehhhhhh
Pl2x3_BP_7	mGTVVIVVSRDERILEELLEVVLKSDPNVKTVRTDDKEKVKEEIEKARKQGRPIVIFIRGATEEVVRDIVEYAQKEGLRVLVIMVDQDQEELERIYEQLKKDGVDVRVTDNEDEAKKRLKELLEKVlehhhhhh
Pl2x3_BP_8	mGTVVIVVSRDEEILERLLKEVLKQDPNVVTVRTDDKEKVKEVIEKAIKQGRPVVIFIRGATADVVRDIVRYLQRQGVRVLVIIVDKDDEELKRIYERLREEGVDVRVTDNEEEAMRRLKDLLQKVlehhhhhh
Pl2x3_BP_9	mGTVVIVVSDDENILEDLLEVVLKDDPNVVTTRVDDKEKVKKWIKEAREKGRPIVVFIRGATREVVREIVEYAQKEGVRVLVIIVDKDDDKLKKIYEQLKKDGVDVRVTDNEDEAKKRLKDLLRKVlehhhhhh
Pl2x3_BP_10	mGTVVIVVSDDEEILEELRKKVEEQDPNVVTTRVDDENKVKEVIEKAIKQGRPVVIFIRGATEDVVKNIVEYLQKQGVRVLVIIVDQDDKKLKRIYDTLKKEGVDVRVTDNRDEAIKRLLELLDKLlehhhhhh
Pl2x3_BP_11	mGTVVIVVSDDENILEDLVEEVRKKDPNIVTTRVDDENKVKELIEKAYKKGRPIVIFIRGATRDVIERIVEELKKRGVRVLVIIVDKDDDKLKKIYKRLQDDGVDVRVTDNREEAIKRLRDLLEKIlehhhhhh
Pl2x3_BP_12	mGTIVIVVSRDENVLEELIKKVLKRDPNVVTTRVDDENKVKKVIERKREEGRPVVIFIRGATREVVRRLVRKLKEEGVRVLVIIVDKDDNELKNIYDELKKEGVDVRVTDNKDEAEKRLLRYLDELlehhhhhh

12 designs for Pl2x3_BP.

R2x3_BP1_A1	mNIVVIVLSNDEESVEKFWEIVKKEGFDVRKVTNTDDLRDDLKDLVEKNNAQFVIVVVSDKEWAERVLKLVKELGRQVIIIFYDDNEERLEKWREEFESKGVTVRRVRTEEEFKKVIEELRKRIgslehhhhhh
R2x3_BP1_A2	mNEVVVILSDNEDVLKKFFEDVKSKGFKVTTAKNLDDAEELLKRLIEEQNVQFVIVVVQDKEWAKRFLERVKEEGRQIIIIFYDTNEEQLEKWREEIESQGVRVRRVRTRDEFQRVLEEIHKEIgslehhhhhh
R2x3_BP1_A3	mNIVVIVLNTNEEELEKFREEVEKKGLTVRTVKTTDEARKLLKKLIEEHNTRFVIVVVSDREWARKFLEEVKREGRQIIIIFYSTNEELLQKFEQDIESQGVRVRRVRTRQEFKKVLEDIEKEIgslehhhhhh
R2x3_BP1_A4	mNIVVVILDTNEDVLKKFLEIVKKQGFTVTRAHTPDRMERELEELIQKHNVQFVIVVVSDKEWAEKALRRVKEQGRQIIIIFYSTNEELLEKWREDIESQGVRVRRVDSPDEFERVLREIEREIgslehhhhhh
R2x3_BP1_A5	mNIVIVVFSTDEETLRKFKDIIKKNGFKVRTVRSPQELKDSIEELVKKYNATIVVVVVDDKEWAEKAIRFVKSLGAQVLIIIYDQDQNRLEEFSREVRRRGFEVRTVTSPDDFKKSLERLIREVgslehhhhhh
R2x3_BP1_A6	mNVVVVVLSTDRRRLEELSDVIKKDGFKVRTVRSPEELKRSIEELLKKYNANFVVVVVDDREFAERAIRFIKEQGATVLIIIYDDNKEQLEEFSEEVERRGFEVRKVDSPDSFKDSLEKLIKEVgslehhhhhh
R2x3_BP1_A7	mRLLVMVFTSDKKLVEEIKREVERRGFRVRDVQSPDELRRDLEKLLREHNAQFVIIVVTDKEWAKEALKKAKEEGVQIFLILWDTNEDELRKFREEVERKGVDVRTATSPDDVKDIVREFLERVgslehhhhhh
R2x3_BP1_A8	mQTFVIVLDTDENSLKKFYQIVKSEGLDVRTVKDPEKAKDLLKDILKKQNIQFVIIVVTDREWAREFLEIAREEGVQIFILIYSDDEDRIEKFSRELSSRGVEVRKVTSPDDFKKVIEEIRERIgslehhhhhh
R2x3_BP1_A9	mLIFVVVLNTNEDDLKKFEEIVKREGLTVRTARDPDKAKDLLKKIVKEQNIQFVIVVVSDKEWAEKMLRVAKEEGVQVIILIYDNNEELLEKFSKELASQGVRVRKVTSPDEFERVIREIRDEIgslehhhhhh
R2x3_BP1_A10	mLIVIVILDTNEKQLRIFLRIIKSKGLQVQIVQSPDKLLQQLKDLAEKQNARVIIIVVRNKELAKKAIELVKEAGFNIFIVIFDTNEEKLRKILEDLQKKGVRLYRVESPQRFKEVIEKIEKELgslehhhhhh
R2x3_BP1_A11	mLIVVVILDTNEEELRKFLQIVKSEGFDVWIWKDPDKALQSLKDVLEKQNAQVVIVVVNNKELAEKALRVIKEVNFEIVIVIFSTNEDDLEKILRDISSRGVKLRRVKTSDEFERVLRELKKEIgslehhhhhh
R2x3_BP1_A12	mLVIVVIVKTDKDILKKLLEKFKSQGFDVRTVKTPEELLKLLRELVEKKNARILVVIVDDENLAELAARRAKDLGVKVIIIIIRQDEEELRKALEEVKRENVTVYVVKDPEKAAEYAIEEVQKIgslehhhhhh
R2x3_BP1_A13	mNVVVIIIDTNEDRLKQFLKIVKSKGFIVRIFKSPKQAIEEAKKIIKKQNATVFIIVVNDEELARKAVDTAKEAGVRIIIIIYSTDENKLKRILKELQSKGVDLRKVKSPDDFKKVLEEIEKEIgslehhhhhh
R2x3_BP1_A14	mNIVVIILDTNREKLELFLKIVKSKGLQVDIFTNPREAIESLEKLIQKQNATVVIVVVNDKELAERFLEVVKKAGLKIILIIYDTDENKLEKILEDLQKQGVELRKVKSPDQFKKVLEELEKEIgslehhhhhh
R2x3_BP1_A15	mPVLVVILSTNEKILREILELVKRKGFTVKIVTSPDLLLKELKRLAEEQNVTSVIIIVNDKELAKKALELVKKLGYNIFIIIIDDNEDDLKKVLEELQKKGVELRKVTSPEDLRKIVKELLERLgslehhhhhh
R2x3_BP1_A16	mRIVVFVLNLDKKLLEEMKREFEEKGIELRSVTNPDDLIKRAEEVVEKKQARVLIVLVNDKDLAKKVAESAKKLGVEVIVVIITTDRREIEELYEELRKENVRVYSFKTPKKAIERIREDAEELgslehhhhhh
R2x3_BP1_A17	mLILIIVLDTDRKRLEIFLKIVKSRGLDVRIFDDPNKAIQSLEDILKKQNVQIVIIVVNDKELAKRFLEVIKKAGAKIIIVIVDDDENKLEKILRDLASKGVQLYKVTSPDQFKNIIEDLDKKIgslehhhhhh
R2x3_BP1_A18	mLVLVVVVSDDRKLLEELLRIVKDKGFIVYISSDPDKALQSLKDLAEDNNVTTIIVIVDDKELAKKALKLIKELGFKIIIIIIDNNKDQVEKILRELAKKGVQLYKVTSPDDAKDILKKLLEELgslehhhhhh

18 designs for R2x3_BP1_A.

R2x3_BP1_B1	mRRLVIVLSTDRKQREEWAKTLRSAGYDVRTVNDPNSATEKLEKILQEREYRIVFIITSDPEVMTRLLETIRKSGSTVLILLYDTDENSLQELVEKLSSAGITVRTVSSPDELLQSFEELSQEIgslehhhhhh
R2x3_BP1_B2	mKFLLIVLSEDNREREEWEETLQSLGYDVRTVSDPESAVRKIREILRRRQLQLVVVLTSDTQVMETLLKTIRRAGSTVFILLKDDDENSLEKVLRRLSSVGIDVDKVSSPDQINESVERFSRKLgslehhhhhh
R2x3_BP1_B3	mKFLLIVKSEDNREREEWEETLQSLGYDVDTISDPESAVRKIREILRRRQLQLVVVLSSDTQFLETLLKTIRRAGSTVFILLKDDDENSLEKVLRRLSSAGIDVDKVSSPDQIQESVERFSRKLgslehhhhhh
R2x3_BP1_B4	mRRLVIVLSTDRKQREELAKTIRSAGYDVRTVNDPNSATEKLEKILQEREYRIVFIITSDPEVMTRLLETIRKSGSTVLILLYDTDENSLQESVEKLSSAGITVRTVSSPDEFLQSFEELSQEIgslehhhhhh
R2x3_BP1_B5	mDVFVMVDTSDEDLVRKIKQLVEDRGYEVRDVRDSDELRKQLEEYLKKENFKKVLIVSSDKNLLKKALEEISRLGYQVFLVLKDQDENELEKFQQEIESKGYEVRKVTDEEEALKVVEEFLKKAgslehhhhhh
R2x3_BP1_B6	mNVFVMVNTSDKDLIEKIKRDVEERGYQVRDVQDENEMKEELKKWLEKQNFKKVLFIVSDSQLAKEVLKIVSELGYQVFLVLKDQDENRLEDFQKEIESKGYEVRKVTDKEEALKIVREFLDKAgslehhhhhh
R2x3_BP1_B7	mDVLVMVNTSNQDLIDKIKDLVENSGYEVRDVQDSNELKDELKRLAEEKNFKKILVIVNDKDLAKKMLELIKKLGYQVFLLLEDKDEDELKKFEQEIRSKGYEVRRVTDEEEALKIVEEFLKKAgslehhhhhh
R2x3_BP1_B8	mEVLVMVNTNNDDLIKKIKQQVKDQGYEVRKVNDEQELEKELKRLAEELNFKKILIISNDKNRLEKMLKRIKELGYQIFLLLEDQDEKELKEFKDRIKSRGYEVRKVTDKDEALKIVKEFLQKAgslehhhhhh
R2x3_BP1_B9	mKILILINTNNDELIKKIKKEVENQGYQVRDVNDSDELKKEMKKLAEEKNFEKILIISNDKQLLKEMLELISKLGYKVFLLLQDQDENELEEFKRKIESQGYEVRKVTDDEEALKIVREFMQKAgslehhhhhh
R2x3_BP1_B10	mDVFVMVNTSDEDLIRKIKRDVEQQGYEVRDVQDSDELRQQLKKWLEEKNFKKVLIISSDENLLREALKIISELGYQVFLLLKDQNEDELERFKNEIESKGYEVRKVTDEEEALKIVRKFLQEAgslehhhhhh
R2x3_BP1_B11	mHYLVIVKSDDENDRKNIWQRISSAGYDVRTVNDPNRATRVIREEWKRHNVQVIFVITSDPNVMKEILRVVKKLGATILILLKDTDEDSLKQLAQDLSSVGIEVRTVSNPDQLRDVFEELSKRAgslehhhhhh
R2x3_BP1_B12	mKYLIIVLSTDEQVRKDIVKKIASKGYDVRTVNDPNNAVDKAKDELEKQNYRVIFIITSDPNVLKDLLRVAEEAGVPVLIIVKDTNEEQLRKLAEELSSAGVEVRTVSSPDEFEKTFKDLSKKAgslehhhhhh
R2x3_BP1_B13	mSYIVIVLTQNEKTRKELAELIKKAGYTVTRVNSPDELSKSIDEAYQKADYRVVVIVVSDPNKLEDAIRLIKDAGAPVLIIVKDQNERRLKEIAKKAKEAGYPVRTVSSPDNFKKTLEELVKRGgslehhhhhh

13 designs for R2x3_BP1_B.

R2x3_BP2_1	mGRVVVVSDDEKVLRKISEELRRQGYEVVQSNDIQKALQIVEKENIRIVIINDKDSNRLSEFIREADRVGVRVLVLSSDENVVKSIQKQWPNARTVRFEDSDDVREAVREFVEQGgslehhhhhh
R2x3_BP2_2	mGKVVFVDQDEEIIRKVAKQMEEDGYEIRTSNDPKEALKRLKRRNIDLIIVNTNDEKLISEVIEEVLKEGAKVLILSSDENIIKQIRQQYPEIEVREANDSDQILKAIKEFVEKGgslehhhhhh
R2x3_BP2_3	mGKVVMVDDDQNILEEVKKRMEEEGYDVQTSNDPQSALDRLKRRNIDMIIISTDDLKKISEVAKEVLEEGAKVLVLTSDEKLKKSVQKQWPKIQTRQASDEEEVKDLVEQFVQEGgslehhhhhh
R2x3_BP2_4	mGKVLFMDDDQEIIEEVAKQMQKDGYEIRTSNDPKEVLDRLKRRNVDMVIINTDDEERISEVAKEVLKEGAKVLVLSSDDKLIESVAKQWPKIDVRKATDKESIKQAVKEFVEKGgslehhhhhh
R2x3_BP2_5	mGKVVFMDDDEEIIREVKKKAEEEGYDIQTSNDPNSVLDRLKRRNIDMIIINTKDEKKISSVAEEVLKEGAKVLVLSSDQNLIEQVAKDWPQIQVREAQDKEQIKDAVEEFIKEGgslehhhhhh
R2x3_BP2_6	mGKILFVSDDQKIIEEVSKKMQKEGYEIRTSNDPKEASKSLSRRNIDLVIILTNDEKLIKELVKKVLEQGADVLVLSSDKKIIEKIRKQYPQIEVRQAQDSEEIKEAVKEFADRGgslehhhhhh
R2x3_BP2_7	mGKVVFLSDDQEIIEEVSKKAEEEGYDIQTSNDKKEIIDRLKRRNIDMIIVKTEDKESISEIIKQVLDSGAKVLILSSDENIIESIRKQYPKVETRRAQDKEEVKDAVEEFLKEGgslehhhhhh
R2x3_BP2_8	mGRVVVMSDDDELLEKVARRLREEGIDVRTSNDPQEALRRMKEQNVRIIIVDDKDEEKISRVIEEADRQGAKMLILTSDERISKSIQEKWPSAQTVRFEDSDDVEELVREFVEKGgslehhhhhh

8 designs for R2x3_BP2.

R3x3_BP1_1	mRKIFVLFNSDPEILEELVKKIREDGVRVILVYSRQDRKEREREIKRFRKKGIDVRTVEDKDKFRDNIREIWQRYPQLDVVIIV<br>TSDNEDVLRDVIEEARKQGVRVFVIYQSKDEERRREAYQRFKSEGVTVRTVRDKEELKEEVERIVRRVgslehhhhhh
R3x3_BP1_2	mSQIFVIFSSDKEILKKIVEELRKRGVRVILVYSDTDRKRREERVEEFRKQGIDVRTVSDKDSFRENIRRIWRRYPQLDVVIIVL<br>TDDEELLEEVIREAREQGVRVVVVYQDDDENERRRAYQRFRSEGVDVRTVSDKEEMKREVERIVERIgslehhhhhh
R3x3_BP1_3	mSKIFVIFSSDPEILKELVKDIRENGVRVILVYRRQDQKDREKWIKEFKKEGVDVRTVQDKEKFRENVREIWERYPQLDVVII<br>VLSDNKDDLEDFIKEARERGVRVIVVYQSKDEERRREAYQEFKSKGVDVRTVSDKEEMKERVKEFVKRVgslehhhhhh
R3x3_BP1_4	mSQIFVIFNSDKDILEELVKQIRDQGVRVILVYWDTDEKRRRERIDEFRKRGIDVRTVEDKEDFRKNVREIWERYPQLDVVII<br>VTTDDEELLKDIIEEAKKQGVRVIVIYQSDDENERREAYQKFRSDGVDVRTVSDKEELKERVKEFVQRIgslehhhhhh
R3x3_BP1_5	mSQIFVVFSSDKDILKRIVKRIREEGVRVILVYRDQDERRRRERLEEFRKQGVDVRTVQDKEQFRENVREIWEQYPQLDVV<br>IIVLSDNKDDIKEFIEEAKKQGVEVYVVYQSDDDDRRKEAEQEFRSKGVDVRTVSDKEELIRQVREFVEKVgslehhhhhh
R3x3_BP1_6	mSQIFVIFSSDPDILKQIVEEIRRKGVRVILVYRRQDRKEREKRLEEFKKKGVDVRTVQDKEQFRRNVQEIWRRYPQLDVVII<br>VLSDNEEDLEEFIREAREEGVRVIVVYQDKDEERRREAYQKFKSRGVTVRTVSDKEEMIREVTRIVRRIgslehhhhhh
R3x3_BP1_7	mSQIFVIFSSDPDILRELVKEIRDNGVRVILVYSDQDRKRREKYLKEFKKEGVDVRTVTDKETFRENVREIWERYPQLDVVII<br>VLSDDREWLKDFIEQARKEGVRVIVVYQDDDDNRRKEAYQEFKSKGVDVRTVSDKEEMKERVREFVKRVgslehhhhhh
R3x3_BP1_8	mSQIFVIFNSNEEILRELVEKIRRNGVRVILVYSSQDEERRRRSIEEFKKEGIDVRTVEDKEQFRENVREIWDRYPQLDVVIIV<br>TTDDKELLKDIIEEAKKQGVEVIVIYQSDDENERREAYQEFRSKGVRVRTVSDEEELKKEVLRFVKEIgslehhhhhh
R3x3_BP1_9	mSQIFVVFSSDPEILKEIVREIKRQGVRVVLLYSDQDEKRRRERLEEFEKQGVDVRTVEDKEDFRENIREIWERYPQLDVVVI<br>VTTDDKEWIKDFIEEAKERGVEVFVVYNNKDDDRRKEAQQEFRSDGVDVRTVSDKEELIEQVRRFVRKVgslehhhhhh
R3x3_BP1_10	mKTVIILVASDKDILEKLFEEFRKRGIKVIVVVKLQDEKKLEKFVKDFRSKGGEVRKVKTDDELLKVLEKIWRENPQWQLYFF<br>VLDDDKNSLKKVIEKLRELGAKVIVIYKDQDDNKLEEVKQEFQSKGVTVKKVTSSEEALKVIKEIVEKIgslehhhhhh
R3x3_BP1_11	mKTIFVIVVSDKNILEEIAKKIKKQGFQVIVVVDVQDEKKLEEFFREFKSKGIQVRKVKTREEAEKWIEKIWREYPQLDLVIIVI<br>SDDKDWLKDFIEKVKELGAQVFVVYKSDDDNKLEKVREEFENQGITVKTVNDDQQAIEVVKEIVEKIgslehhhhhh
R3x3_BP1_12	mQQIFVFVASDENLLKEFAKYVDQQGARVIIIVDVQDKKKLEKFVENIKSQGFEVRKAEDSKKVLEIIKKIYEEKPQLQIYIIV<br>VTDDENLLEKVIKLAKDLGVQVIVVYKAQDDNKLKKILQKFKSEGIDVKTVDSDQQALQLIKEIVEKLgslehhhhhh

12 designs for R3x3_BP1.

R3x3_BP2_1	mgKKIVVFDDDDEILKKLVKKIRELGIEVYLLFKNEDEQELKEIIKKLRSDGFNVRKVNDVEDAKEWVERINVETLIILTDDEEIIRKLIEKAEKLGIEIMVLIKNQDEDELEKLQNNLSSQGIEVRKVSDRNELIQIIEEFVKKLgslehhhhhh
R3x3_BP2_2	mgKKIVVFDDDDQILKEIVEQIRRLGIEVYLLLKNKDDQELEELIKKLRSDGVEVRKVNDVEDAKDWVEKINVDILIILTDDEKIIRELIKKARKLGIEILVLIENQDEKELEELQNELSSQGIEVRKVSDKNELIQIIREFVEKLgslehhhhhh
R3x3_BP2_3	mgQKVVVFDDDDEILQKLVEKIRRLGIEVYLLLKNDDEQQLEELIKKLRSDGIEVRKVEDVEDAKEWIKRINVDILIILTDDEKIIEELIRLAQKLGIQIMVIIKNQDEDSLEKIQNKLSSKGIEVRKVSDKQQLIDIVEEFVRKIgslehhhhhh
R3x3_BP2_4	mgKKIVVFDDDDEILKKLVKKIRELGIEVYLLLKNDDDKELQELIDKLRSDGINVREVNDVEDAKDWIEKINVDILIIITDDKQIIKELIEKARKLGIEILVIIKNQDEDKLEKLQNELKSKGIEVRKVSDRNELIQIIKEFVEKIgslehhhhhh
R3x3_BP2_5	mgKKIVVFDDDDEILKKLVEQIRDLGIEVYLLLKNDDEKQLEQLIKELRSDGINVRKVNDVEDAKDWVEKINVETLIIITDDKKIIRELIKKAQKLGIEIMVIIKNQDENDLEKLQNELSSKGIEVRKVSDRDQLIKIVKKFVKELgslehhhhhh
R3x3_BP2_6	mgQKVVVFSTDKEILKKLVKRIRELGIEVYLLFKNKDDQELDDIIKDLRSDGFNVRKVNDVEDAKDWVEKINVDILIIITSDEKIIRELIKKAQKLGIEIMVIIRNQDEDELEKLQNKLSSQGIEVRKVSDRNQLIDIIKKFIEEIgslehhhhhh
R3x3_BP2_7	mgQKVVVFSSDKDILRELVRRIRELGIEVYLLLKNDDDKQLEQIIKELRSKGFNVRKVNDVDDAVKWVRKINVDTLIIITNDEKIIRKLIEEAKKLGIEIMVIIKNQDEKELEELQNKLSSEGIEVRKVSDSQELIDIIKKFIEKIgslehhhhhh
R3x3_BP2_8	mgQKVVVFSSDDEILKKLVKKIRELGIEVYLLLKNDDEKELKKLIQELRSDGFNVREVNDVEDAKDWVEKINVTNLIIITDDKKIIEELIKKARKLGIEIMVIIKNQDENELEKLQNELSSKGIEVRKVSSREELIRIVEKFVREIgslehhhhhh
R3x3_BP2_9	mgQKIVVFSTDDEILEKLVRTIRDLGIEVYLLLKNDDDQELEDLIKKLRSEGINVRKVDDVDDAKEWVERINVDILIIITNDKQIIEELIKKAKKLGIEIMVIIENQDENELEKLQNELSSKGIEVRKVSDRNQLIQIIEDFIKKIgslehhhhhh
R3x3_BP2_10	mgQKVVVFSSDDEILKKLVQRIRDLGIEVYLLLKNEDDDELDDLIKELRNDGINVREVNDVDDAKDWVEKINVDVLIIITDDEKIIEELIKKAQKLGIEIMVIIKNQDEKELQELQQKLSSKGIEVRRVSDRDELIEIIREFIEKIgslehhhhhh
R3x3_BP2_11	mgKKVVVYSSDDEILERIVERIKELGIEVYLLLDNKDKKQLEELLKRLRSQGFNVRQVNDVNDAEDWIEKVNVDIVVIITNDEEIIRKLIEKAKELGVQILVIYKNDDKDQLEQIRKDLESEGIEVREVSDKQKLIELVEKFIEKIgslehhhhhh
R3x3_BP2_12	mgDKIVVFSTDDEILERLAEQIRRLGVEVYLLLKNDDDQELDDLIKRLRSQGIEVRKVNDVDDAIDWVKKIGVNILIIITSDEKIIRKLIEAARKLGIEIMVIIENQDEDELEKLQNDLSSQGIEVRKVSDRQELIQIIRDFIEKLgslehhhhhh
R3x3_BP2_13	mgRKIVVFSSDDEILRELVRQIRELGIEVYLLLSNKDDQELDDLIKKLRSKGINVRKVNDVEDAKDWVNKIGVDILIIITNDEKIIEELIKEAKKLGIEIMVIIENQDEKQLQELQQKLSSKGIEVRRVSSRDELIDIIKDFVKKLgslehhhhhh
R3x3_BP2_14	mgTKIVVFSTDDRILRELVRQIRDLGIEVYLLLKNEDDQELEDLIKSLRSQGVEVRKVDDVDDARDWIKKIGVDIVIIITDDEKIIRELIKEAQKLGVEIMVIIKNQDENKLEKLQNELSSKGIEVRKVSDEEQLIKIVKDFVKKLgslehhhhhh
R3x3_BP2_15	mgTKIVVFSTDDEILKKLAEKIRKLGVEVYLLLKNDDEQELQDLIDKLRSQGIEVREVKDVNDAIEWVKRIGVTNLIIITDDEKIIEELIKAAEKLGIEIMVIIKNKDEDELEKLQNKLSSKGIEVRKVSDRQELIEIIEKFIEELgslehhhhhh
R3x3_BP2_16	mgKKIVVFSTDDEILKKLVKKIRDLGIEVYLLLKNDDDEELQKLIDKLRSEGIEVREVKDVEDAKDWIEKSGVDVLIIITDDEKIIKELIKKAEELGIEIMVVIKNKDEDELEKLQNELSSKGIEVRKVSDKDELIKIVEQFVKDLgslehhhhhh

16 designs for R3x3_BP2.

R3x3_BP3_1	mgRILVIVSTDKNQIRRLIELAKKLGIQQIIVVTKDDSLLDEARKAGAERVFLSKDDEKLIRLLIKLGVQILITDNDKIMLRLAEELQNVIVLSKDQEALKRVKKQLENAKVFEITDDNELLKIVEEIswglehhhhhh
R3x3_BP3_2	mgQIFVVITNDEQRIEDMWELAKRIGFTRIVIITSDERLKDRAEELGADRIILLTDREKMIEEAKKLGADIIITNNEEIARRIAEKDKNLIILTEDKNAVENVQKDISDARIFEIQTKERIREILEKLgslehhhhhh
R3x3_BP3_3	mgRIIVIIVTDEQKIEDMWEILKEIGVDRIVIITSNKQLAERAKELGVDRIFLLTDDELIAEIVKKLGADIVFSENRDIAKKIIRKLKNIIILSNDEQLVKELQKEASDARVFNVQTKQDFKDLIEKIgslehhhhhh
R3x3_BP3_4	mgRIVVVFVTDKQKLRDVWEIMEEAGVDRRIVITSDEELAKEAKKLGVDRIFLLTDVEQAAEIAKKLGVDLVFSEDEDFLKKVVRKLKNVIILSNDKQIVEELQKDISDARVFNVSTKEDLREIVRKIgslehhhhhh
R3x3_BP3_5	mgNIIVVFVTDKQKLEDVWEIMKEVGVDRIVILTSDEELRKKAEKLGADRIFLITDEEKAAELAKKLGVDLVFSENEKFLKKVIKKLKNVIILSNDTRIVEELQKEISDARVFNVETKDKLKELLERIgslehhhhhh
R3x3_BP3_6	mgKIVVVVVTDKQKLEDVWELLKRIGFDRIIIITSDKELLEKAKELGVDRIFLITNKELAAEIAKKLGVDIVFSENREIAKRIIRKLKNAIILSNDTELVEELQKDASDARVFDINTKEDLREILEKLgslehhhhhh
R3x3_BP3_7	mgRIIVIFVTDKQKLRDMWEILKEIGVDRIVILTSDEELAKEAKRLGVDRIRLLTDDQQLAEIAKQLGVDIVFSENERFLKEVARKLKNAIILSNDQRIVEELQKEISDARVFQVSTKQDFKDLIEKIgslehhhhhh
R3x3_BP3_8	mgRIVVVFVTDRQKLKDIWELLEEIGVDRIIILTSDKELKEEAEKLGADRIFLITDEELAARLAKDLGVDIVITENKKFAKKVIKELKNVIILSNDTELVEELQKEISDARVFDIETKDKLKELLERIgslehhhhhh
R3x3_BP3_9	mgRIFVVVVTDEQKLEDMWELAERVGVDRIIIITSDEKLVKKAEKLGVDRIFLLTDKELAAEIAKRLGVDIVFSENREIAKRIIRKLKNAIVLSNDKELVEELQKDASDARVFNVSTKEDARELIERIgslehhhhhh
R3x3_BP3_10	mgRIIVVFVTDKQKLEDLWRILKEIGVDRIVILTSDEELLKKAKKLGADRIFLIKDEEIAARIAKDLGVDIVFSENKRFLEEVIRELKNVIILSNDTEIVEELQKRISDARVFDINDKQDFKDLIEKIgslehhhhhh

10 designs for R3x3_BP3.

SI References

S1. Lin YR*, et al.* Control over overall shape and size in de novo designed proteins. *Proceedings of the National Academy of Sciences of the United States of America* **112**, E5478-5485 (2015).

S2. Ho BK, Thomas A, Brasseur R. Revisiting the Ramachandran plot: hard-sphere repulsion, electrostatics, and H-bonding in the alpha-helix. *Protein science : a publication of the Protein Society* **12**, 2508-2522 (2003).

S3. Wang G, Dunbrack RL, Jr. PISCES: a protein sequence culling server. *Bioinformatics* **19**, 1589-1591 (2003).

S4. Tyka MD*, et al.* Alternate states of proteins revealed by detailed energy landscape mapping. *J Mol Biol* **405**, 607-618 (2011).

S5. Park H*, et al.* Simultaneous Optimization of Biomolecular Energy Functions on Features from Small Molecules and Macromolecules. *J Chem Theory Comput* **12**, 6201-6212 (2016).

S6. Marcos E*, et al.* Principles for designing proteins with cavities formed by curved beta sheets. *Science* **355**, 201-206 (2017).

S7. Bhattacharya A, Tejero R, Montelione GT. Evaluating protein structures determined by structural genomics consortia. *Proteins* **66**, 778-795 (2007).

S8. Huang YJ, Powers R, Montelione GT. Protein NMR recall, precision, and F-measure scores (RPF scores): Structure quality assessment measures based on information retrieval statistics. *Journal of the American Chemical Society* **127**, 1665-1674 (2005).

S9. Hyberts SG, Goldberg MS, Havel TF, Wagner G. The solution structure of eglin c based on measurements of many NOEs and coupling constants and its comparison with X-ray structures. *Protein science : a publication of the Protein Society* **1**, 736-751 (1992).
