# Supplementary material for: Role of backbone strain in de novo design of complex α/β protein structures
Source: Nat Commun. 2021 Jun 24;12:3921. doi: 10.1038/s41467-021-24050-7 (PMC8225619; doi:10.1038/s41467-021-24050-7)
Supplement: Supplementary file 2 — Peer Review File [file 41467_2021_24050_MOESM2_ESM.pdf]

REVIEWER COMMENTS

Reviewer #1 (Remarks to the Author):

In the current manuscript, Koga et al. applies the protein design procedure previously developed in their group (Rosetta algorithms), which has been successfully applied to small alpha/beta folds, to larger and more complex alpha/beta folds, i.e. Ploop and Rossman folds with 5 and 6 beta-strands. To validate the success or failure, for every designed protein a series of properties were examined: expression, solubility, CD spectra typical of alpha-beta proteins, stability (Tm), monomeric state and well resolved 1H, 15N-HSQC. The NMR 3D structure was determined for three selected designed proteins, which behave satisfactorily at all the previous tests. Experimental and designed structure was identical for the Ploop blueprint, but the experimental structures of the two Rossman fold blueprints showed a strand swap. The possible reasons for this failure are analysed and new designs are done and experimentally examined. It is found that the overall backbone strain, which is not considered within the design principles, should be taken into account in the case of large proteins.
I find that the manuscript is solid and well-written, and of merit to be published at the journal Nature Communications. It might be published as it is, but I suggest the authors the following minor changes to improve it
1. I think that to include a Figure showing a flowchart of the tests performed for validating the protein designs would be helpful for following the description in the main-text. In my opinion, it would be clearer that only the well-behaved proteins at one test go to the next one.
2. In Figure 3, I consider that including a ribbon representation of the NMR structures below the ensembles would facilitate the comparison between the designed and the NMR structures for the three initial blueprints.

Reviewer #2 (Remarks to the Author):

I appreciate the intent of this study, which is to design increasingly sophisticated alpha/beta folds using a series of design rules developed by Koga et al. A series of Rossmann-like and P-loop like folds are designed, synthesized and characterized both biophysically and in many cases by solution NMR. Several structures match the models, supporting the overall design approach. However, a few designs have structures with a strand-swap. The origin of this swap is unclear as Rosetta energy calculations favor the target fold over the observed one. Thus a challenge is proposed - to identify the nature of problem - is it one of kinetic trapping of a misfolded intermediate, or are there issues with the energy function and/or sampling protocol?

This is a fascinating problem and I can imagine that proteins are a tensegrity network of noncovalent interactions and as more complex structures are proposed, design shortcomings may propagate through this network and lead to unintended features. However, even after multiple readings, I was a little lost as to the direction of the investigative process and experimental design. If the goal of the work is to augment the design principles of previous work, then this needs to be clarified. I do not think additional work is needed, but the work as it is needs to be made more accessible.

(1) The Thr->Asp mutation is intended as a negative design strategy to bury a charge in the off-target state and shift the free energy minimum toward the target. Was this position chosen rationally or through a computed mutagenesis scan? It would be helpful if this was clarified. The outcome of a molten globule could be interpreted as the authors suggest - but it could also be other things. That said, it suggests that the issue may be with folding - either due to kinetic traps or some energetic aspect of structure the current score does not address.

(2) It is proposed that doing sequence-independent folding and scoring for occupancy of key main-chain hbonds in the ensemble can be used to identify which elements of structure may be relating to

strain. It is perhaps here where I am the most confused. In the folding approach, are new terms included that allow the investigators to converge on a modified backbone with favorable properties (shorter strands, effective terminal packing)? Also, it is not clear why these features - and not others, or the entire structure overall, is the issue with the target fold. There is clearly a lot of intuition from the investigators that allowed them to develop this protocol and identify these factors - but it is not clear how general / automatable they are.

In the end, there is resolution - a design with the target topology is achieved. The iterative application of design and structure determination is well highlighted. This work is very important and should be published here. The main issues are with helping the reader understand the path the researchers took to get the solution.

reviewed by Vikas Nanda

Reviewer #3 (Remarks to the Author):

This manuscript by Koga et al reports the Rosetta design of αβ proteins containing 5 or 6 β-strands. Two common topologies, the P-loop (Pl) fold and the Rossmann fold, are designed by adding αβ units onto previous designs of αβ proteins with 4 strands (Koga et al Nature 2012). The goal is to extend design to larger proteins, of a size more typical of functional protein domains; this is a major and very challenging goal. While success is achieved, at a rate of ~12%, for the PI fold after the first round of design, the first 3 Rossman fold designs do not adopt the intended topology, instead exhibiting swapping of strands in the center of the β-sheet to form a structure like the PI-fold. The paper includes substantial modelling analysis, and a second round of design produces the intended Rossman folds. This study includes much work - in addition to the design and analysis, it includes expressing and characterizing ~89 designs, including determining NMR solution structures of 6 designed proteins. Based on analysis of the designed proteins, the failure of the initial Rossmann fold designs is attributed to frustration in the β-sheet and poor packing of the N and C terminal helices. Overall, this is an extensive and important study, which could be an important advance in the field.

My main reservation concerns the explanation of the results, which appear unsystematic and not fully convincing. A key failing is that the Rosetta energies for the first round Rossmann fold designs are lower than the corresponding experimental (PI) folds. The Discussion considers a range of possible contributing factors, but there is no definite explanation. Furthermore, how β-sheet hydrogen bonding and C-terminal helix packing (among many other possible factors) were identified as the source of design failure is not specified. Such information on the reasons for success or failure, and how to identify them, is critical for guiding future designs. Other specific points to address are given below.

1. Analyzing hydrogen bond formation probability for the β-sheet is logical to assess how well defined the design is in this region, and it might provide a measure of fold “frustration” when hydrogen bonds are not well formed. On the other hand, for the first round designs the low probability H bonds are concentrated at the ends of the strands (Fig 5), similar to the shorter strands in the second round designs. The sum of the log of H bond probabilities will give particularly negative scores for bonds that are not formed (Fig 5); but, otherwise, the total H bond formation spanning the sheet looks higher in the Rossmann designs than the corresponding experimental structures. Additional explanation and justification of the H bonding analysis is needed. Could the preceding considerations be related to the Rosetta energy discrepancies?

2. Visual inspection of the first round structures (Fig 3) obviously suggests a problem with packing of the N and C terminal helices in the core for R2x3_BP1_A5 and R2x3_BP1_B9. However, other packing might also be lacking. Can the relative importance the terminal helix packing relative to other packing or stabilizing effects be defined? Were these helices focused on because they are relatively long-range interactions and so particularly likely to contribute to folding co-operativity?

3. The authors report Tm values for thermal unfolding of the proteins, and comment on the co-operativity of unfolding. The fitting equation, other fitted parameters (and error estimates), heating rate and its influence on results, reversibility of unfolding, and a reference should be addressed. The unfolding enthalpy or heat capacity might give further insight into how well folded the proteins are. These properties are pertinent because the high Tm does not necessarily give a good measure of stability as it can be caused by low cooperativity and corresponding low change in heat capacity of unfolding.

4. Please clarify the rationale for engineering curvature. Is curvature more pronounced and are strands shorter in natural proteins that adopt these folds than in the first-round designs? Ref 7 Koga et al 2012 noted "the extent of folding to a particular motif is very strongly dependent on the lengths of the secondary structures", how does this study agree, differ, or go beyond the previous finding? Much work from the Baker group has emphasized consistency between local and non-local interactions, how does this study reveal how to identify the inconsistency? Is there related information in the packing density, and does the density increase with curvature?

5. It would be valuable to include more complete information:
-Rosetta scores of design models R2x3_BP4_7 and R3x3_BP_3 and the corresponding NMR structures (as in Supp Fig 6 for first round designs).
-H bonding probabilities for PL2x3_BP NMR in Fig 5
-Bar graphs for H bond probabilities (as in Fig 5) for the designs in Fig 6.

6. There appear to be quite extensive errors in the references e.g. ref 5 is cited when it should be ref 6, 6 for 7, 9 for 10.

To Reviewer #1:

To validate the success or failure, for every designed protein a series of properties were examined: expression, solubility, CD spectra typical of alpha-beta proteins, stability (Tm), monomeric state and well resolved 1H, 15N-HSQC. The NMR 3D structure was determined for three selected designed proteins, which behave satisfactorily at all the previous tests.

1-1) I think that to include a Figure showing a flowchart of the tests performed for validating the protein designs would be helpful for following the description in the main-text. In my opinion, it would be clearer that only the well-behaved proteins at one test go to the next one.

As you pointed out, we performed the tests sequentially so that only the well-behaved proteins at one test go to the next one. To clearly show this point, we modified Supplementary Tables 2-8 on the results of experimental tests and added the description on this point to the legend.

1-2) In Figure 3, I consider that including a ribbon representation of the NMR structures below the ensembles would facilitate the comparison between the designed and the NMR structures for the three initial blueprints.

Following the reviewer's suggestion, we added a ribbon representation of the NMR structures in Figure 3 (and Figure7) to make the strand swap more visible and facilitate the comparison.

To Reviewer #2:

2-1-1) The Thr->Asp mutation is intended as a negative design strategy to bury a charge in the off-target state and shift the free energy minimum toward the target. Was this position chosen rationally or through a computed mutagenesis scan? It would be helpful if this was clarified.

We chose this position by visual inspection, comparing the design model in the target state and the NMR structure in the off-target state. We clarified this in the section "Origin of strand swapping" in the Results (p. 6).

The origin of this swap is unclear as Rosetta energy calculations favor the target fold over the observed one. Thus a challenge is proposed - to identify the nature of problem - is it one of kinetic trapping of a misfolded intermediate, or are there issues with the energy function and/or sampling protocol?
2-1-2) The outcome of a molten globule could be interpreted as the authors suggest - but it could also be other things. That said, it suggests that the issue may be with folding - either due to kinetic traps or some energetic aspect of structure the current score does not address.

As you pointed out, there is a possibility that the Rossmann fold design model conformations and the observed strand swapped P-loop conformations could have roughly similar free energies, with the latter favored due to kinetic accessibility or a small free energy advantage, as described in the text. However, we concluded that this possibility is unlikely, because the buried charged residue would strongly disfavor the swapped state and proteins would be difficult to be kinetically trapped at the swapped state during the time for NMR structure determination. We modified the section "Origin of strand swapping" in the Results.

2-2-1) It is proposed that doing sequence-independent folding and scoring for occupancy of key main-chain hbonds in the ensemble can be used to identify which elements of structure may be relating to strain. It is perhaps here where I am the most confused. In the folding approach, are new terms included that allow the investigators to converge on a modified backbone with favorable properties (shorter strands, effective terminal packing)?

No new energy terms were included to obtain a modified backbone. The modified backbone structures (that is, blueprints) without backbone strain were identified by trial and error, in which the compatibility of each considered blueprint with its global tertiary structures is evaluated by generating backbone structure ensembles. In the near future, this process should be automated. We added a sentence into the last of the second paragraph in the Discussion (p. 12, the last line).

If the goal of the work is to augment the design principles of previous work, then this needs to be clarified. I do not think additional work is needed, but the work as it is needs to be made more accessible.
2-2-2) Also, it is not clear why these features - and not others, or the entire structure overall, is the issue with the target fold. There is clearly a lot of intuition from the investigators that allowed them to develop this protocol and identify these factors - but it is not clear how general / automatable they are.

In our previous studies, we discovered the design rules relating local backbone structures (a few consecutive secondary structures and connecting loops) to supersecondary structure motifs with helices packed on paired β-strands, which were originated from local backbone strain. In this work, to solve the unintended strand swapping, we hypothesized that global backbone strain arising from incompatibility between the supersecondary structure motifs and a global tertiary structure should be considered. Thus, we investigated the incompatibility in terms of β-sheet hydrogen bonding and packing between the terminal helices zipping the overall structure, as the cause of design failure. To

clarify this, we modified the Introduction (p. 3, l. 6), the section "Compatibility of blueprint with global tertiary structure" in the Results (p. 7, middle), and the first and second paragraphs in the Discussion.

To Reviewer #3:

My main reservation concerns the explanation of the results, which appear unsystematic and not fully convincing. A key failing is that the Rosetta energies for the first round Rossmann fold designs are lower than the corresponding experimental (PI) folds. The Discussion considers a range of possible contributing factors, but there is no definite explanation.

We agree with you, but it is difficult to identify the exact defects and improve the energy function, or consider configurational entropy as discussed in the Discussion. In the near future, we hope to achieve some solutions by improving the energy function to generate less strained backbone structures.

Furthermore, how β-sheet hydrogen bonding and C-terminal helix packing (among many other possible factors) were identified as the source of design failure is not specified. Such information on the reasons for success or failure, and how to identify them, is critical for guiding future designs.

Thank you for the comments. In our previous study, we discovered the design rules by seeking the consistency between local backbone structures and supersecondary motifs with α-helices packed on paired β-strands, based on folding simulations and analyses of naturally occurring protein structures. In this work, to solve the unintended strand swapping, we hypothesized that the consistency can be applied in the next level structure hierarchy, the relation of supersecondary motifs to global tertiary structures. Thus, we investigated the inconsistency in terms of β-sheet hydrogen bonding and terminal helix packing zipping the overall structure, as the cause of design failure. To clarify this, we modified the Introduction (p. 3, l. 6), the section "Compatibility of

blueprint with global tertiary structure" in the Results (p. 7, middle), and the first and second paragraphs in the Discussion.

Other specific points to address are given below.

3-1) Analyzing hydrogen bond formation probability for the β-sheet is logical to assess how well defined the design is in this region, and it might provide a measure of fold "frustration" when hydrogen bonds are not well formed. On the other hand, for the first round designs the low probability H bonds are concentrated at the ends of the strands (Fig 5), similar to the shorter strands in the second round designs. The sum of the log of H bond probabilities will give particularly negative scores for bonds that are not formed (Fig 5); but, otherwise, the total H bond formation spanning the sheet looks higher in the Rossmann designs than the corresponding experimental structures. Additional explanation and justification of the H bonding analysis is needed. Could the preceding considerations be related to the Rosetta energy discrepancies?

As you pointed out, the Rossmann designs contains a greater number of hydrogen bonds than the corresponding NMR structures. This is one of the reasons that the Rosetta energies for the first round Rossmann-fold designs are lower than those of the corresponding experimental folds. However, we rather focused on hydrogen bonds formed with low probabilities and thus, we took the sum of the log of hydrogen bond probabilities. The low probability hydrogen bonds were concentrated at the ends of the strands likely due to the inherent twisting of parallel β-strands. The twisting makes β-strand pairs being separated at the ends for the longer strands. To clarify this, we modified the second paragraph in the section "Compatibility of blueprint with global tertiary structure" in the Results (p. 7, the last).

3-2) Visual inspection of the first round structures (Fig 3) obviously suggests a problem with packing of the N and C terminal helices in the core for R2x3_BP1_A5 and R2x3_BP1_B9. However, other packing might also be lacking. Can the relative importance the terminal helix packing relative to other packing or stabilizing effects be defined? Were these helices focused on because they are relatively long-range interactions and so particularly likely to contribute to folding co-operativity?

In Fig. 3, we showed that designs and NMR structures in the Rossmann fold have different order of strands, not the difference of packing. To make the figure easier to understand, we modified it. We agree with you about the reason on the importance of the terminal helix packing.

3-3) The authors report Tm values for thermal unfolding of the proteins, and comment on the co-operativity of unfolding. The fitting equation, other fitted parameters (and error estimates), heating rate and its influence on results, reversibility of unfolding, and a reference should be addressed. The unfolding enthalpy or heat capacity might give further insight into how well folded the proteins are. These properties are pertinent because the high Tm does not necessarily give a good measure of stability as it can be caused by low cooperativity and corresponding low change in heat capacity of unfolding.

Following your suggestions, we investigated the reversibility of unfolding for all proteins shown in Figs. 2 and 7. R2x3_BP4_7 is reversible at the end temperature of unfolding transition, but other designed proteins are not, which prevents from obtaining thermodynamic properties such as unfolding enthalpy or heat capacity. Therefore, we deleted the descriptions on the cooperativity and indicated the temperature at the midpoint of the transition as Tm, which was obtained by fitting with a sigmoidal function. We described the details in the section "Circular dichroism (CD) spectroscopy" in the Methods.

3-4) Please clarify the rationale for engineering curvature. Is curvature more pronounced and are strands shorter in natural proteins that adopt these folds than in the first-round designs?

We considered that the origin of the strand swapping is the incompatibility between the backbone blueprints and global tertiary structures. Engineering the β-curvature used in this work is one of the approaches to solve the incompatibility of terminal helix packing. Naturally occurring proteins should achieve the compatibility by using other ways, for example, the elongation of loop lengths, although the quantitative analysis is difficult due to the structural complexity.

Ref 7 Koga et al 2012 noted "the extent of folding to a particular motif is very strongly dependent on the lengths of the secondary structures", how does this study agree, differ, or go beyond the previous finding? Much work from the Baker group has emphasized consistency between local and non-local interactions, how does this study reveal how to identify the inconsistency?

Thank you for the comments. This study agrees with the previous finding because the lengths of the secondary structures in the blueprints were determined based on the previous rules. Furthermore, we extended the consistency to global tertiary structures. How to identify the inconsistency was described in the above response.

Is there related information in the packing density, and does the density increase with curvature?

Following your comments, we added one figure as Supp Fig. 14.

3-5) It would be valuable to include more complete information:

-Rosetta scores of design models R2x3_BP4_7 and R3x3_BP_3 and the corresponding NMR structures (as in Supp Fig 6 for first round designs).

Following your comments, we added one figure as Supp Fig. 16.

-H bonding probabilities for PL2x3_BP NMR in Fig 5

PL2x3_BP NMR has the same blueprint as PL2x3_BP design, thus H bonding probabilities will be the same.

-Bar graphs for H bond probabilities (as in Fig 5) for the designs in Fig 6.

Following your comments, we added one figure as Supp Fig. 15.

3-6) There appear to be quite extensive errors in the references e.g. ref 5 is cited when it should be ref 6, 6 for 7, 9 for 10.

We appreciate you pointing this out. We modified them.

REVIEWERS' COMMENTS

Reviewer #1 (Remarks to the Author):

The minor points I raised on the original version of the manuscript have been properly addressed. I consider the manuscript acceptable for publication.

Reviewer #2 (Remarks to the Author):

This revision clarifies my issues with the first draft and I have no further changes to suggest. The study complements previous work, showing the value of structural determination in refining computational design methodologies.

Reviewer #3 (Remarks to the Author):

The authors' responses and revisions reasonably address important points raised in review and improve the manuscript by adding useful information for others to build on this substantial and important research.

To Reviewer #1:

The minor points I raised on the original version of the manuscript have been properly addressed.

I consider the manuscript acceptable for publication.

We deeply appreciate your comments and suggestions.

To Reviewer #2:

This revision clarifies my issues with the first draft and I have no further changes to suggest. The study complements previous work, showing the value of structural determination in refining computational design methodologies.

We deeply appreciate your comments and suggestions.

To Reviewer #3:

The authors' responses and revisions reasonably address important points raised in review and improve the manuscript by adding useful information for others to build on this substantial and important research.

We deeply appreciate your comments and suggestions.
